# Supplementary material for: Synthesis of Carbosilane and Carbosilane-Siloxane Dendrons Based on Limonene
Source: Polymers (Basel). 2022 Aug 12;14(16):3279. doi: 10.3390/polym14163279 (PMC9416742; doi:10.3390/polym14163279)
Supplement: Supplementary file 1 [file polymers-14-03279-s001.zip › polymers-1810903-supplementary.pdf]

SUPPORTING INFORMATION

CONTENT

SI1. NMR spectra

SI2. GPC curves

SI1. NMR spectra

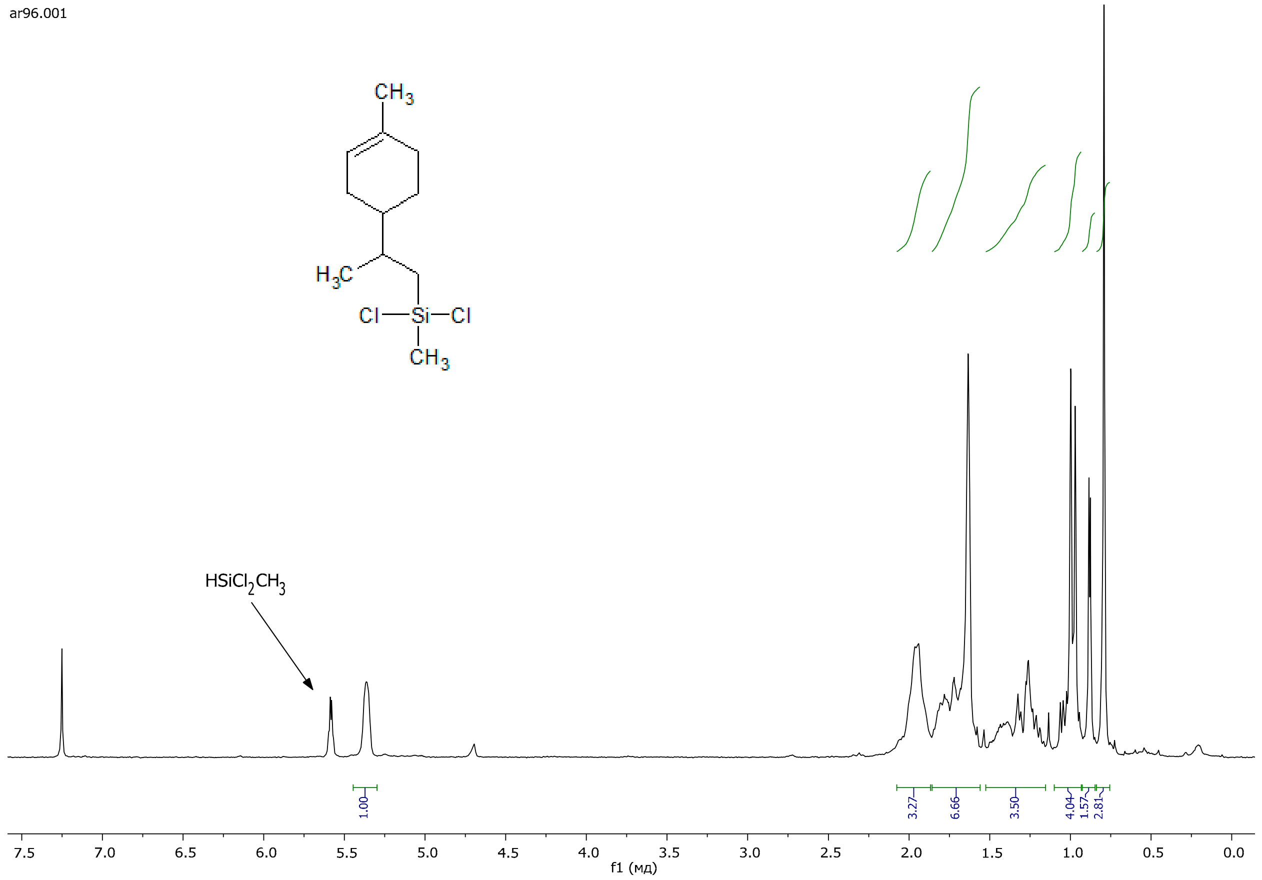

Figure S1. ^{1}H NMR spectrum of Dichloromethylsilyllimonene (Lim-G_0Cl^2).

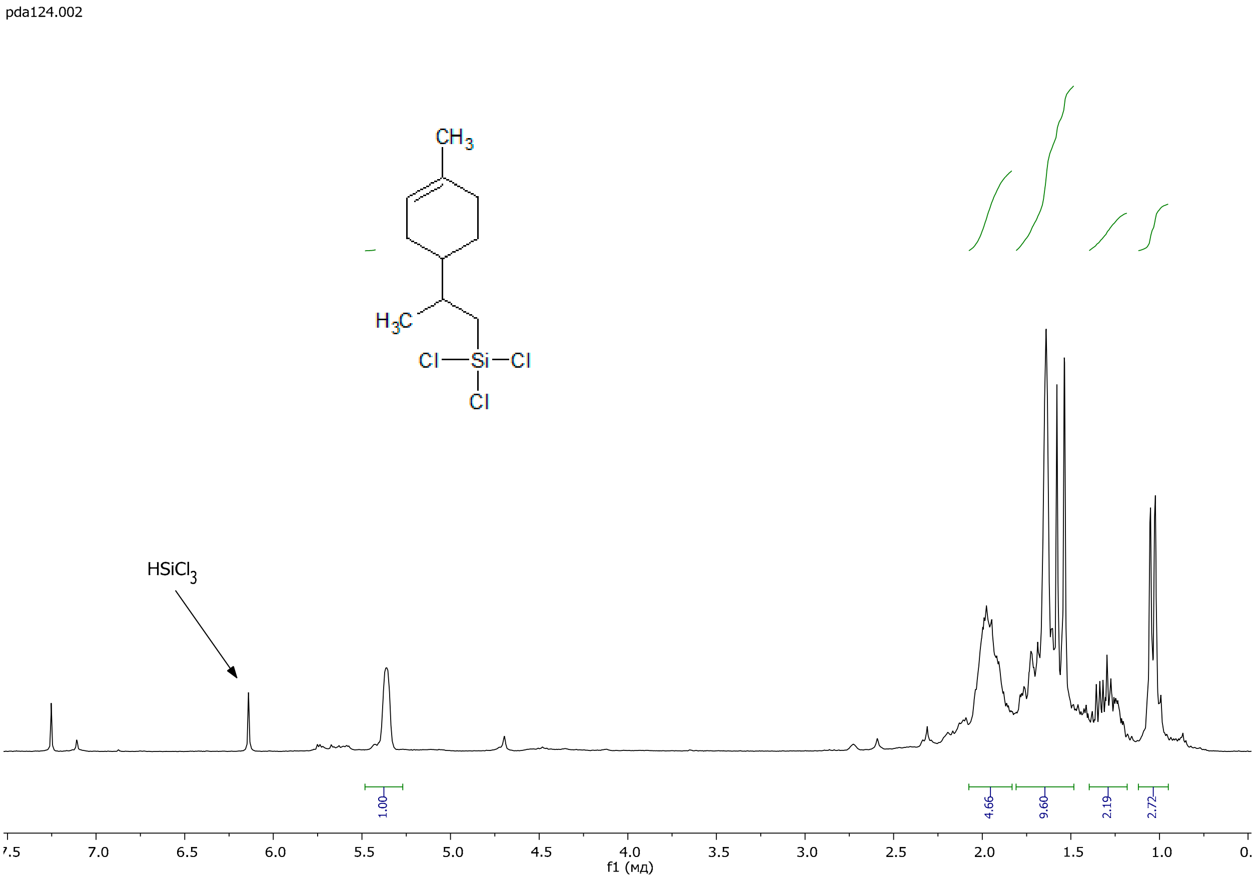

Figure S2. ^{1}H NMR spectrum of Trichloromethylsilyllimonene (Lim-G_0Cl^3).

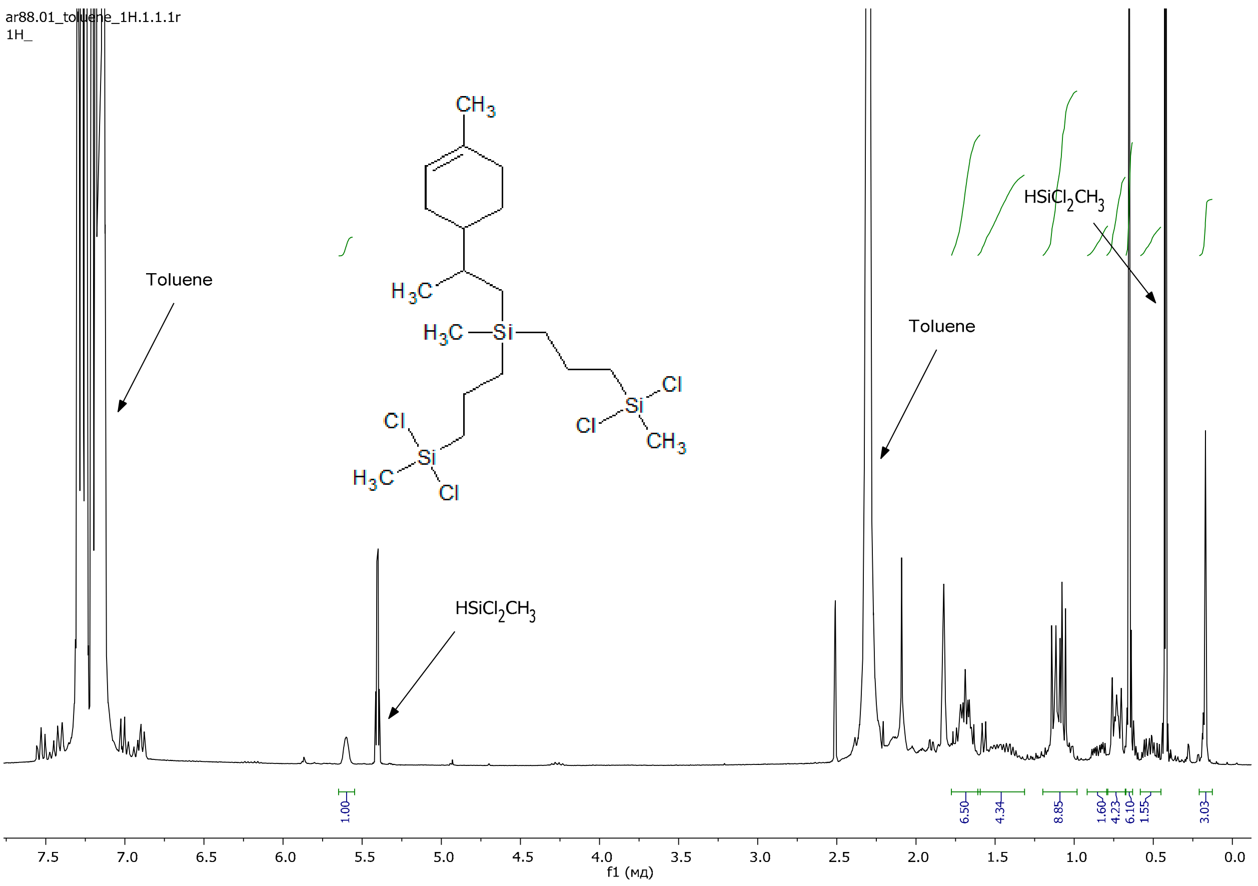

Figure S3. ^{1}H NMR spectrum of Lim-G_1Cl^4.

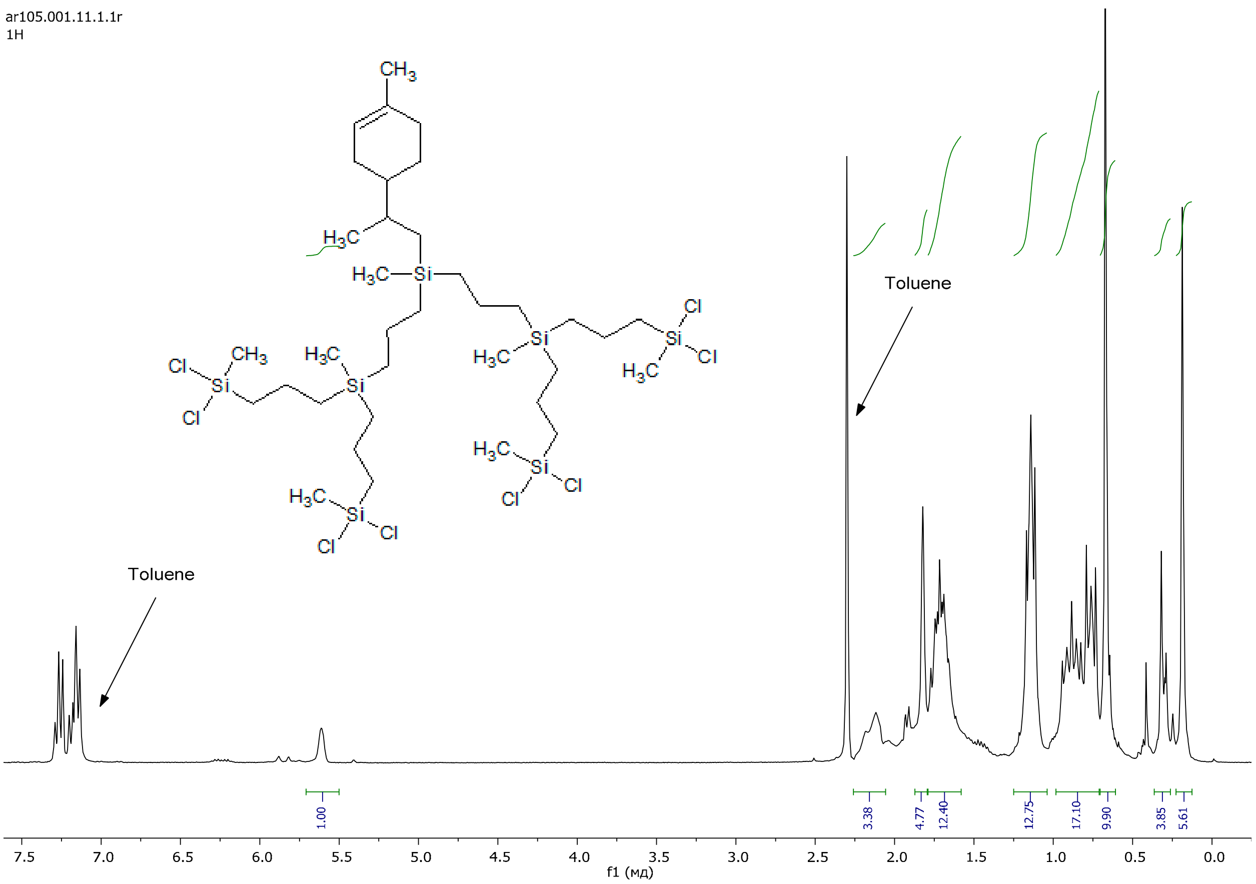

Figure S4. ^{1}H NMR spectrum of Lim-G_2Cl^8.

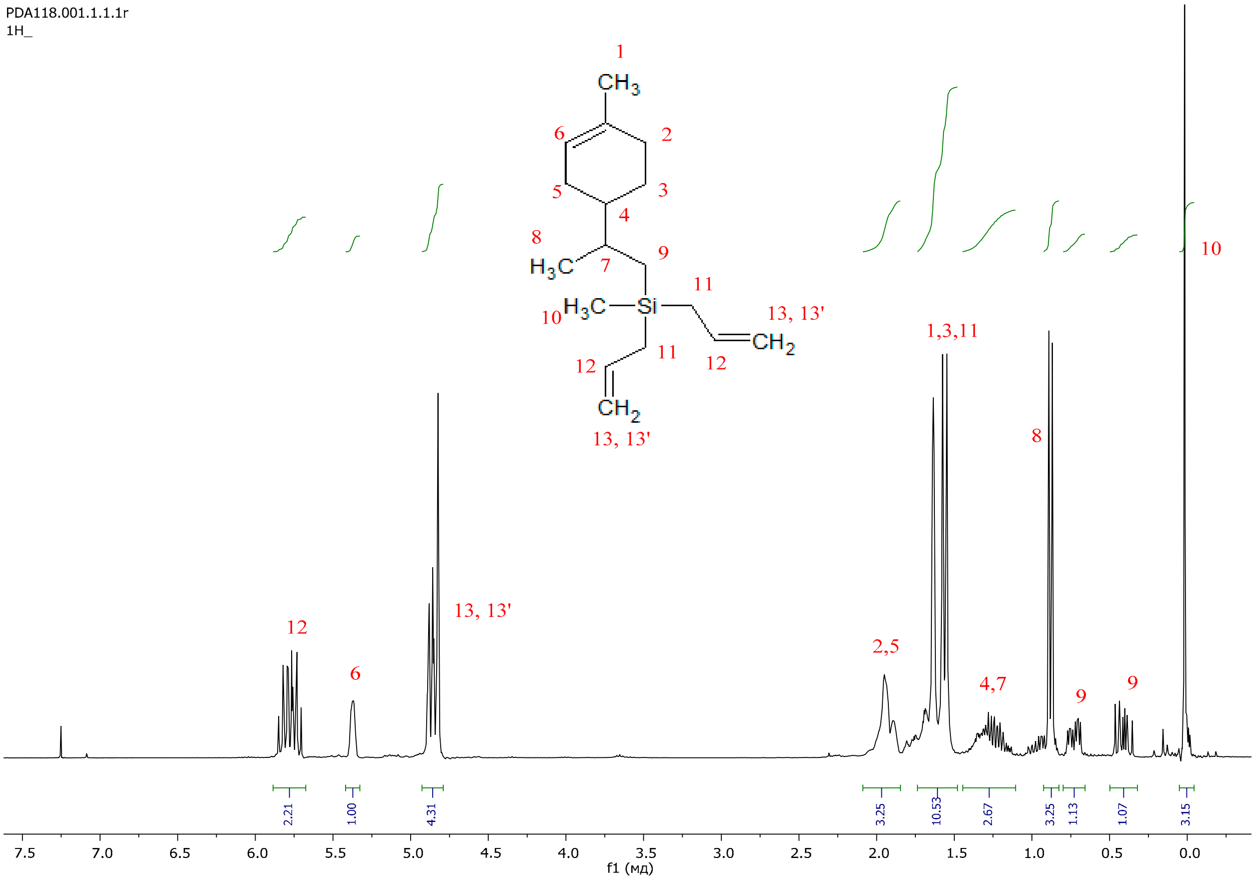

Figure S5. ^{1}H NMR spectrum of Diallylmethylsilyllimonene (Lim-G_0All^2).

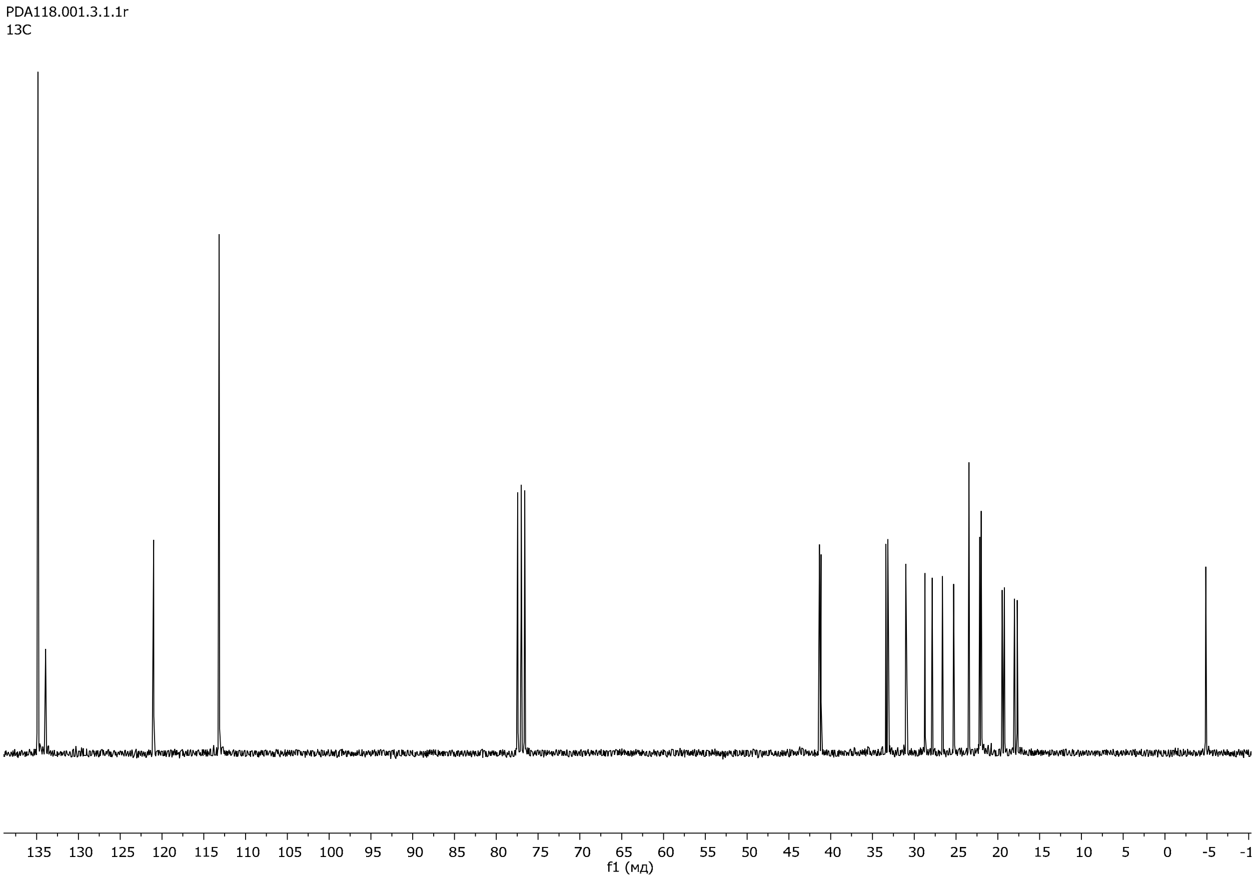

Figure S6. ^{13}C NMR spectrum of Diallylmethylsilyllimonene (Lim-G_0All^2).

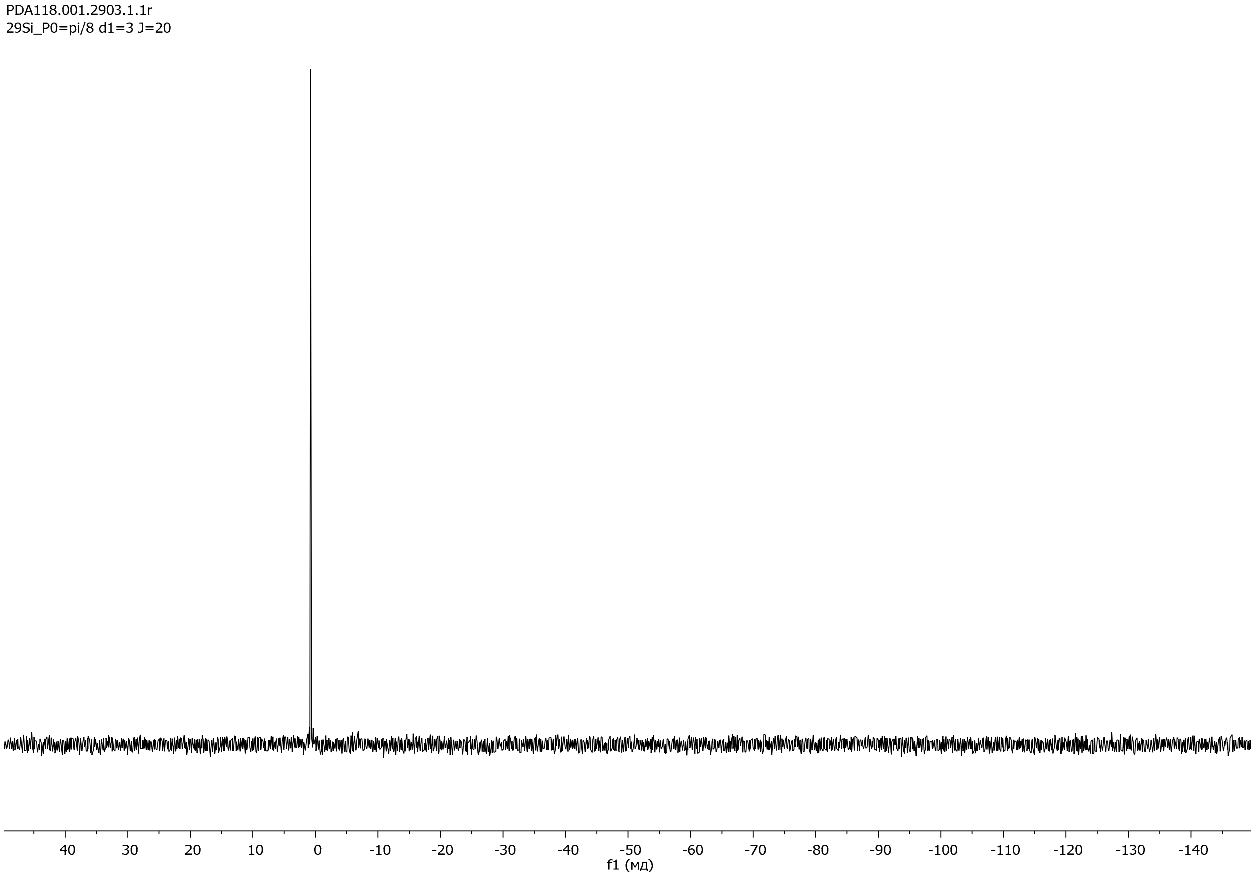

Figure S7. ^{29}Si NMR spectrum of Diallylmethylsilyllimonene (Lim-G_0All^2).

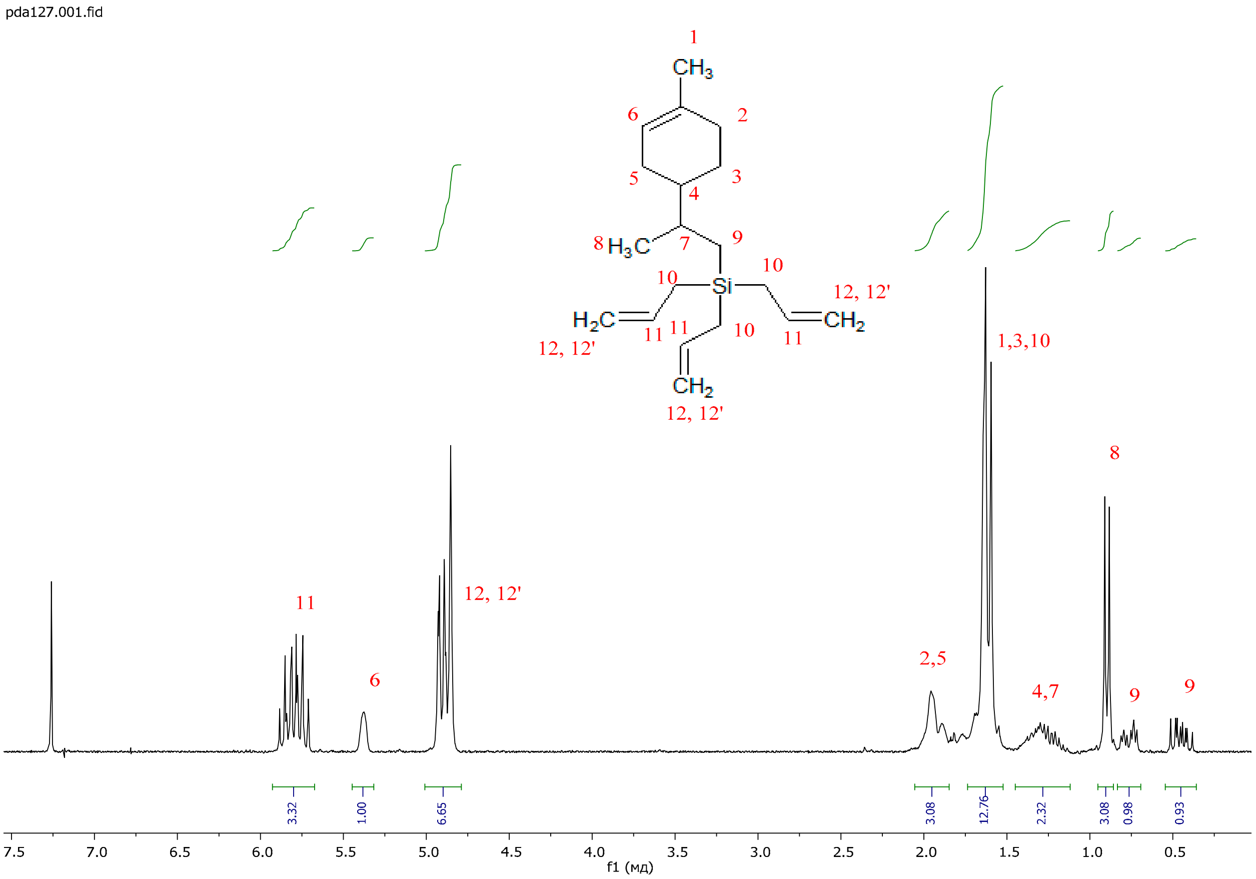

Figure S8. ^{1}H NMR spectrum of Triallylsilyllimonene (Lim-G_0All^3).

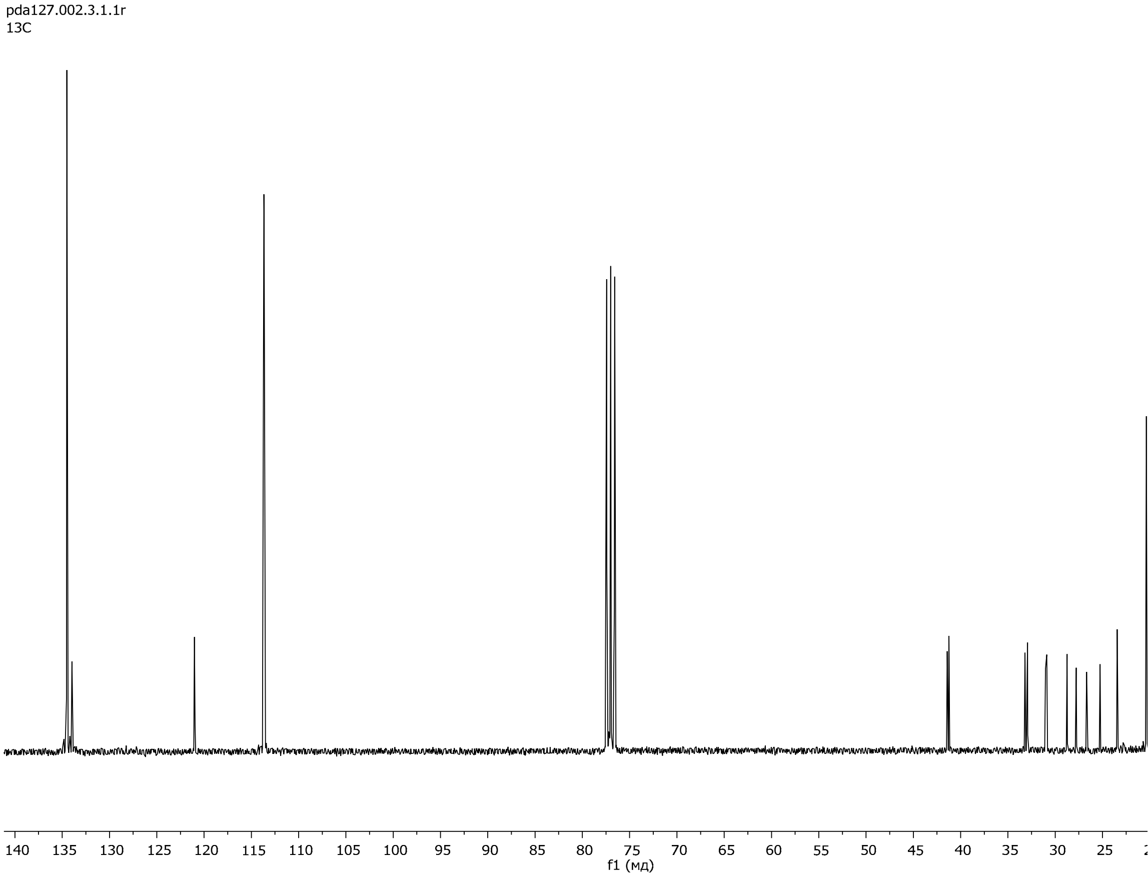

.

Figure S9. ^{13}C NMR spectrum of Triallylsilyllimonene (Lim-G_0All^3).

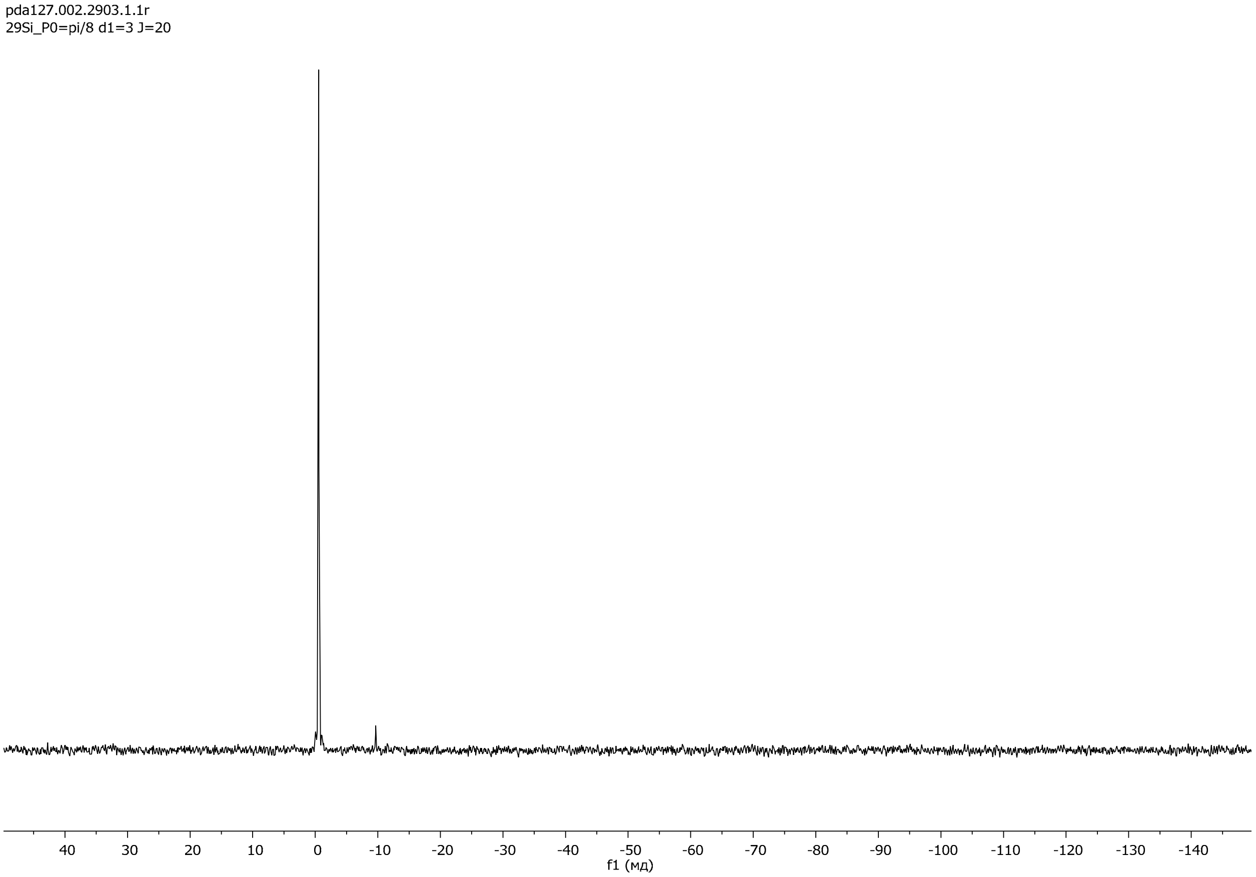

Figure S10. ^{29}Si NMR spectrum of Triallylsilyllimonene (Lim-G_0All^3).

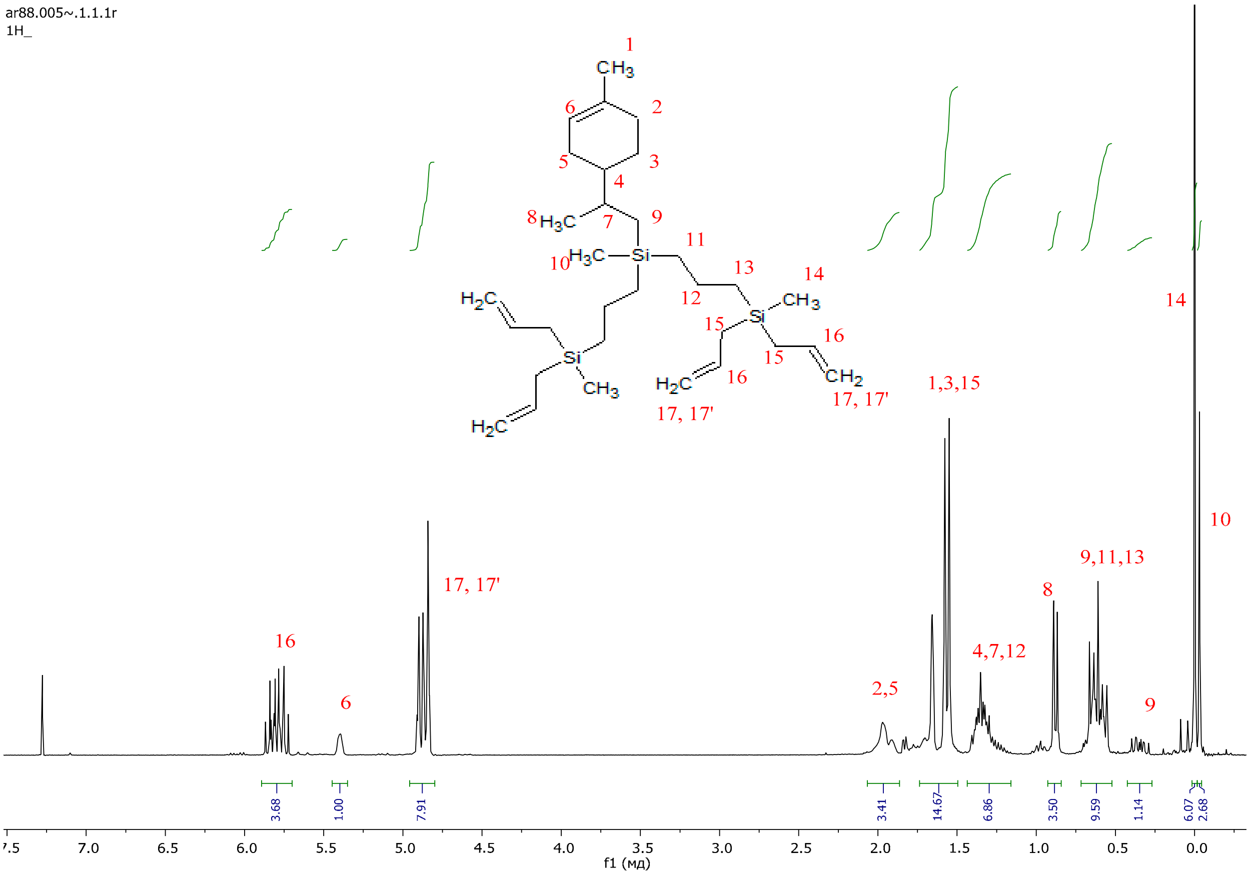

Figure S11. ^1H NMR spectrum of Lim-G_1All^4.

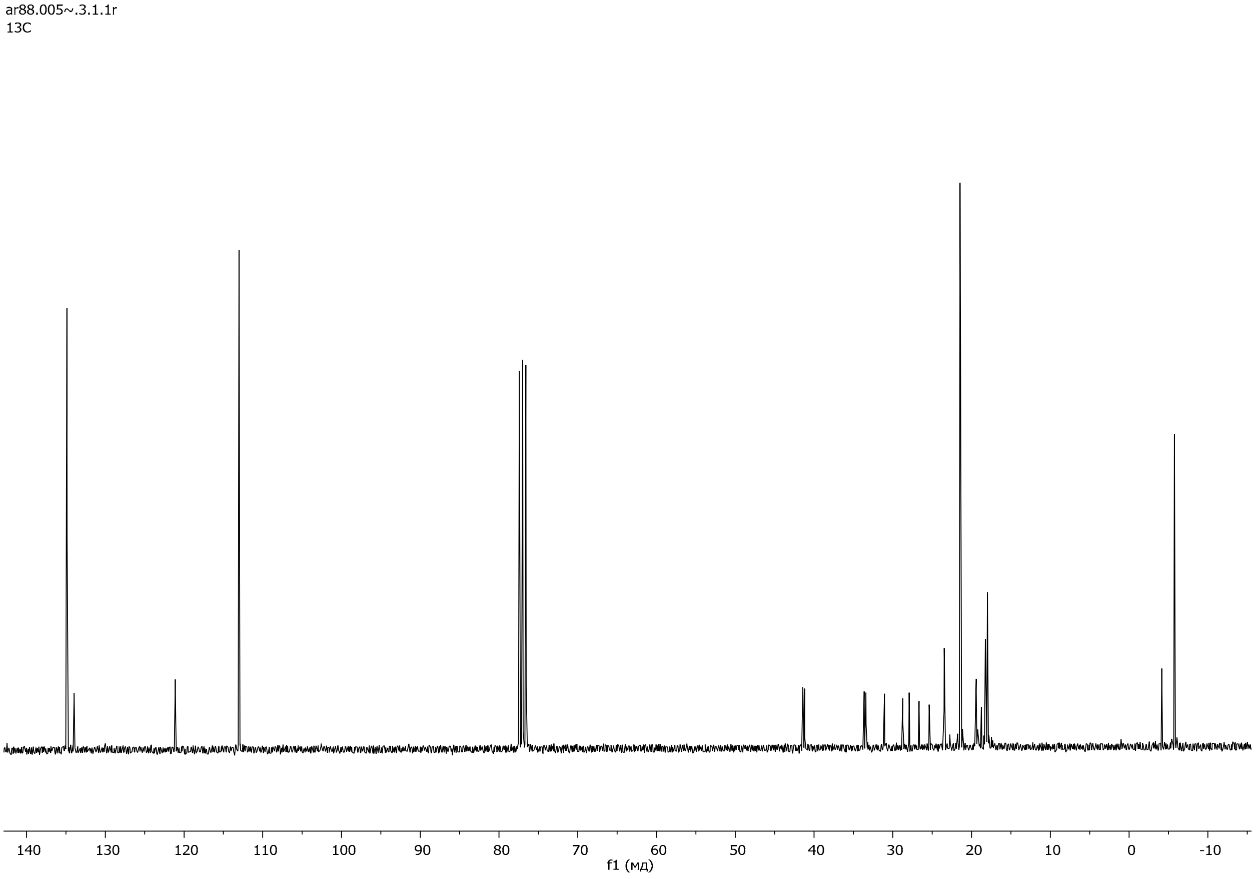

Figure S12. ^{13}C NMR spectrum of Lim-G_1All^4.

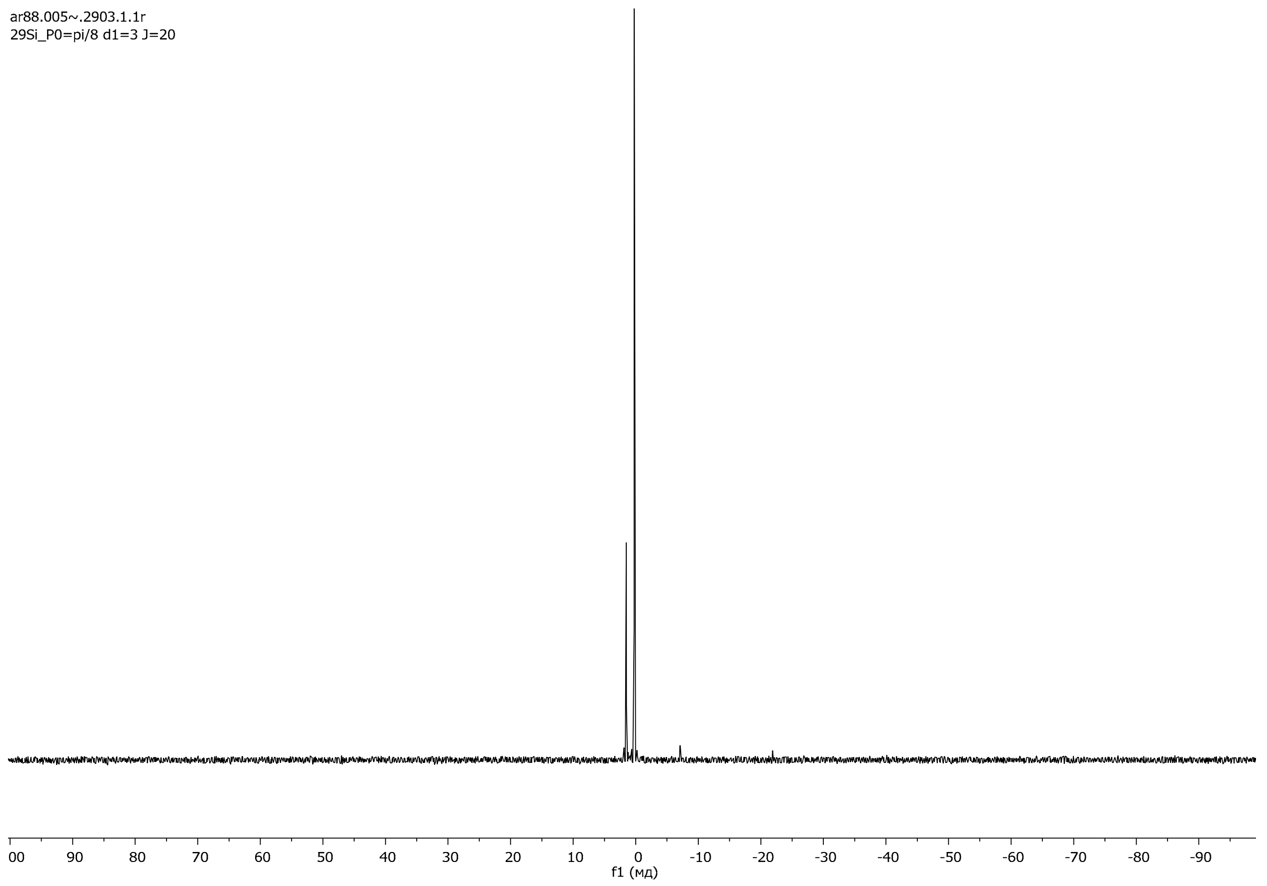

Figure S13. ^{29}Si NMR spectrum of Lim-G_1All[4].

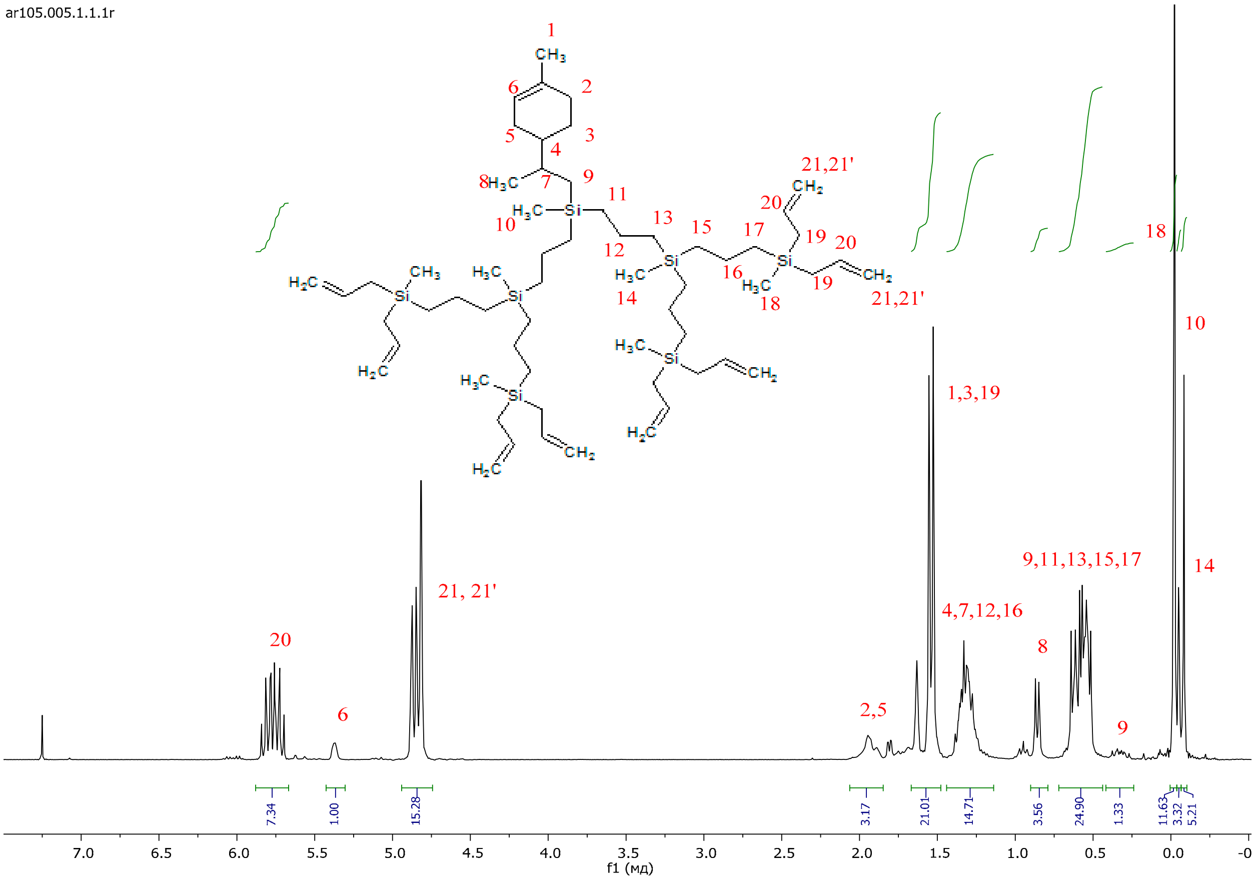

Figure S14. ^{1}H NMR spectrum of Lim-G_2All^8.

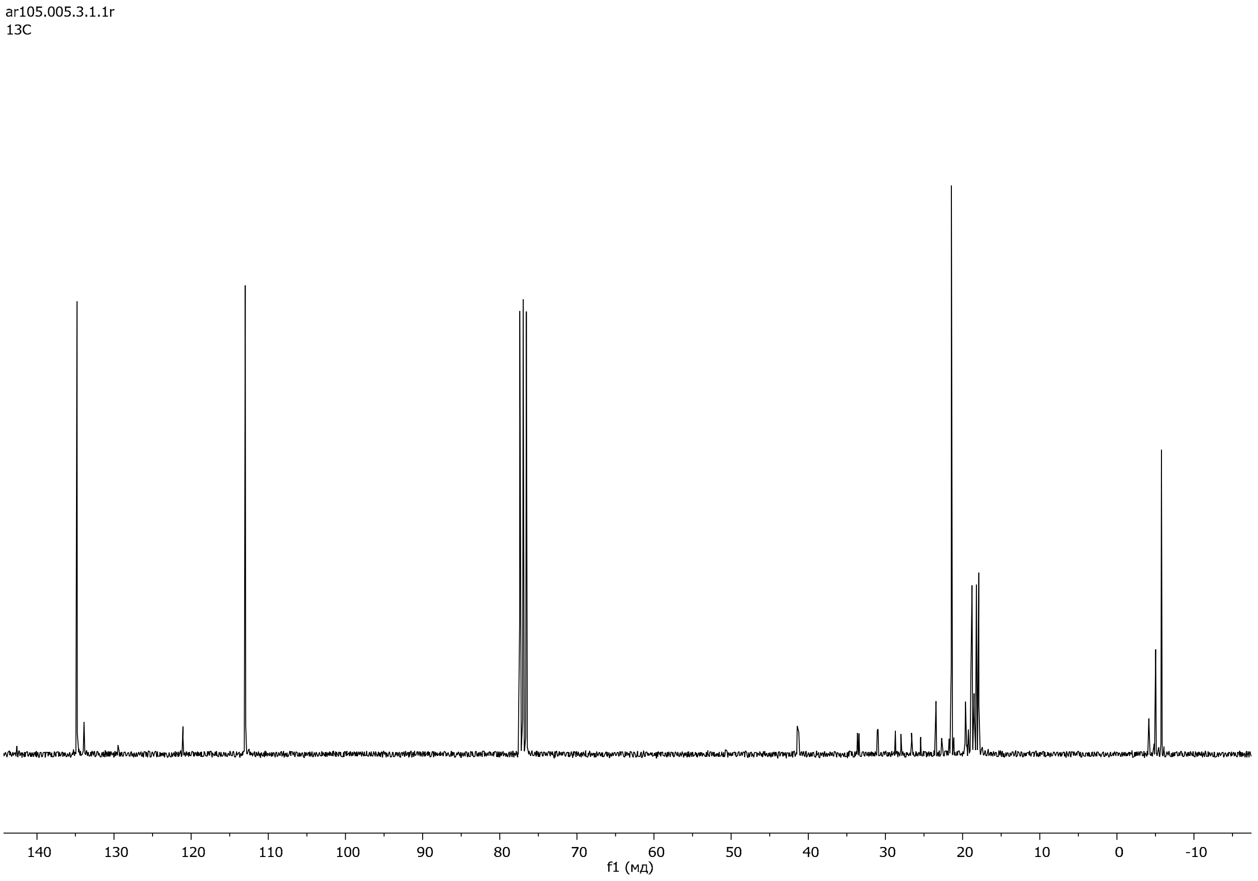

Figure S15. ^{13}C NMR spectrum of Lim-G_2All^8.

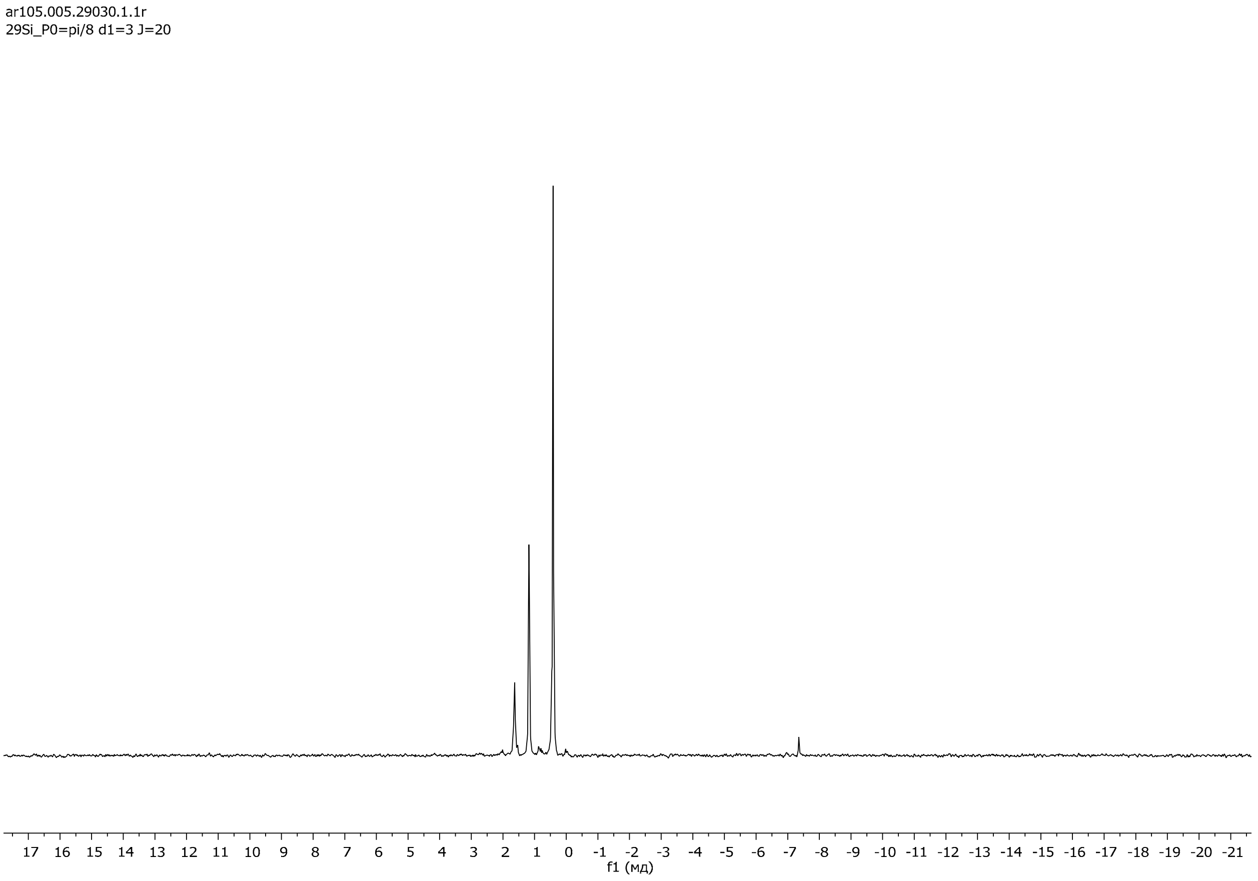

Figure S16. ^{29}Si NMR spectrum of Lim-G_2All^8.

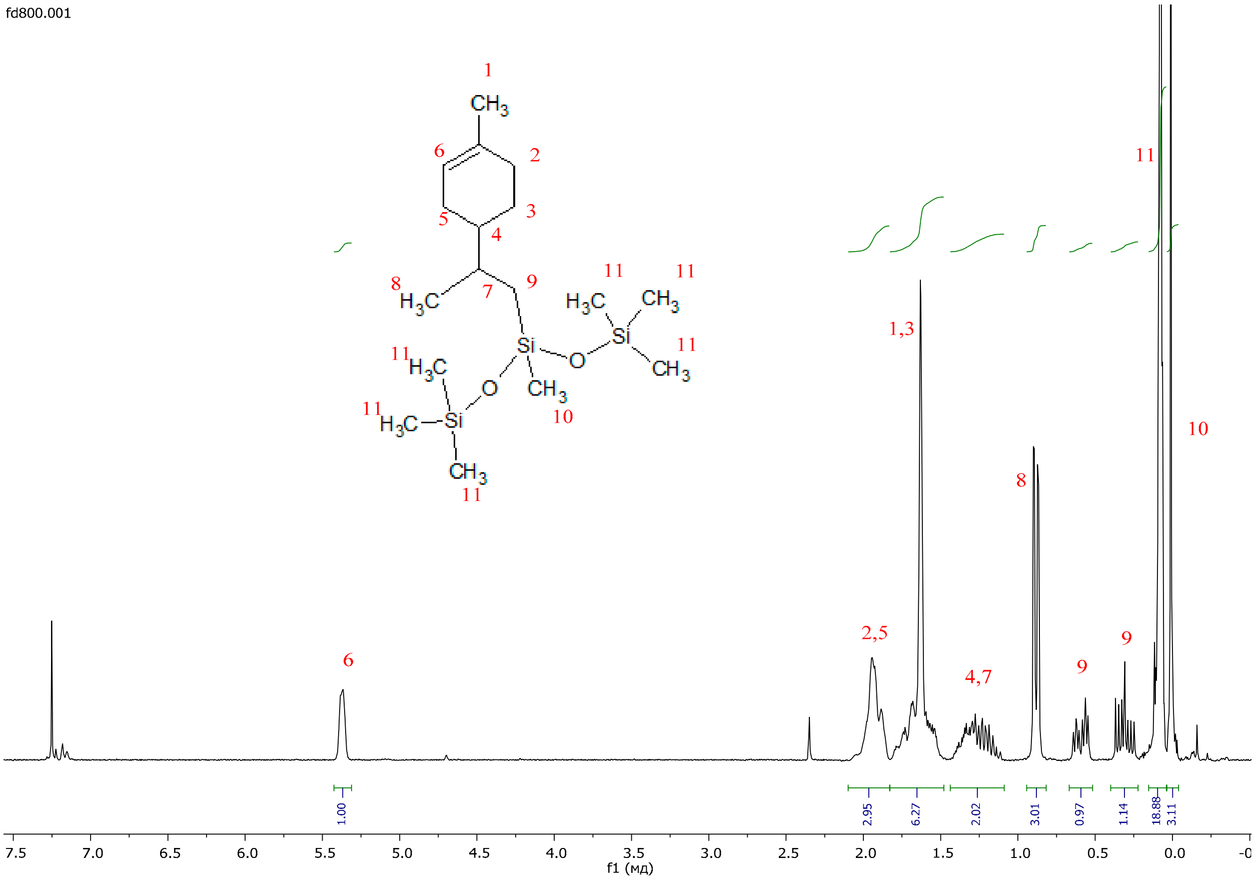

Figure S17. ^{1}H NMR spectrum of Heptamethylsilyllimonene (Lim-$G_{0,5}$TMS^{2}).

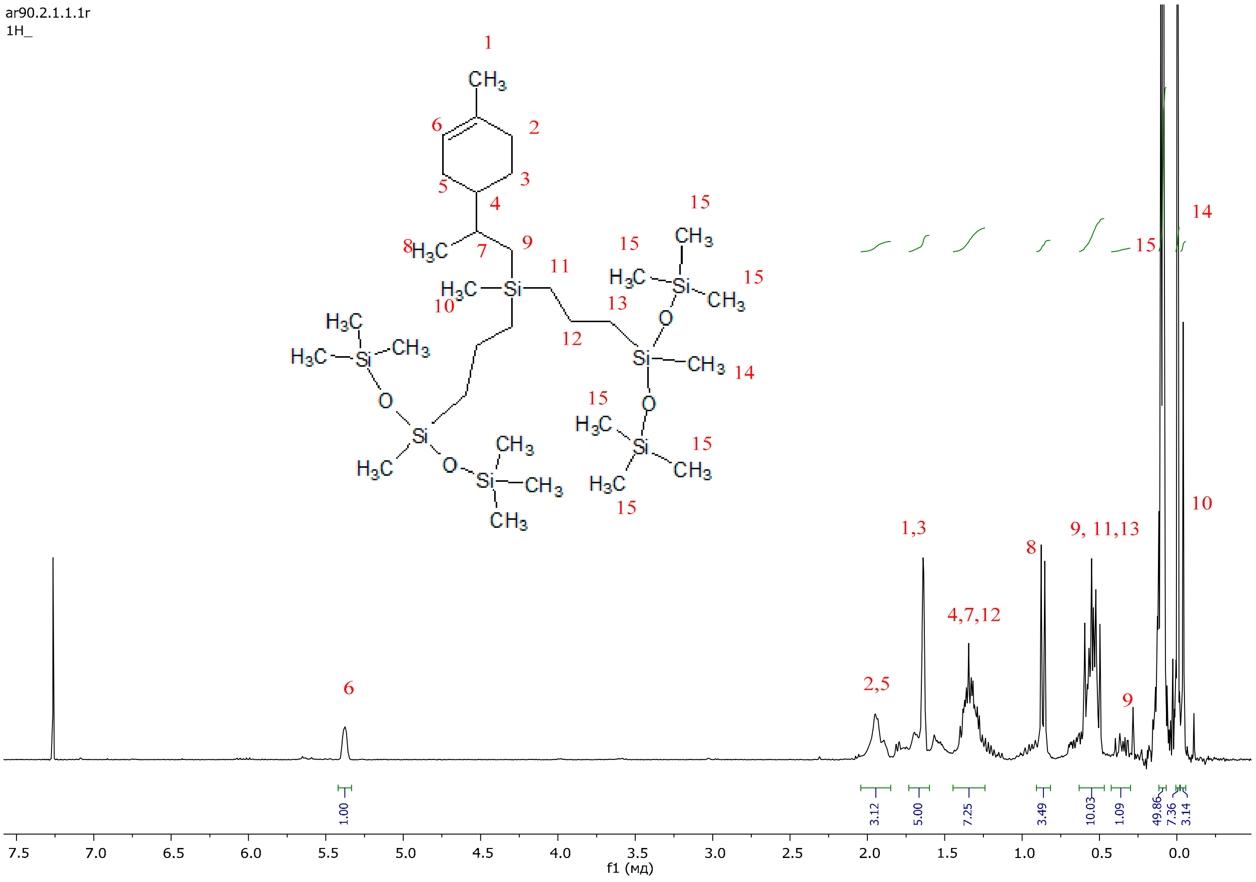

Figure S18. ^{1}H NMR spectrum of bis(heptamethylsilylpropyl)methylsilyllimonene (Lim-$G_{1,5}$TMS^4).

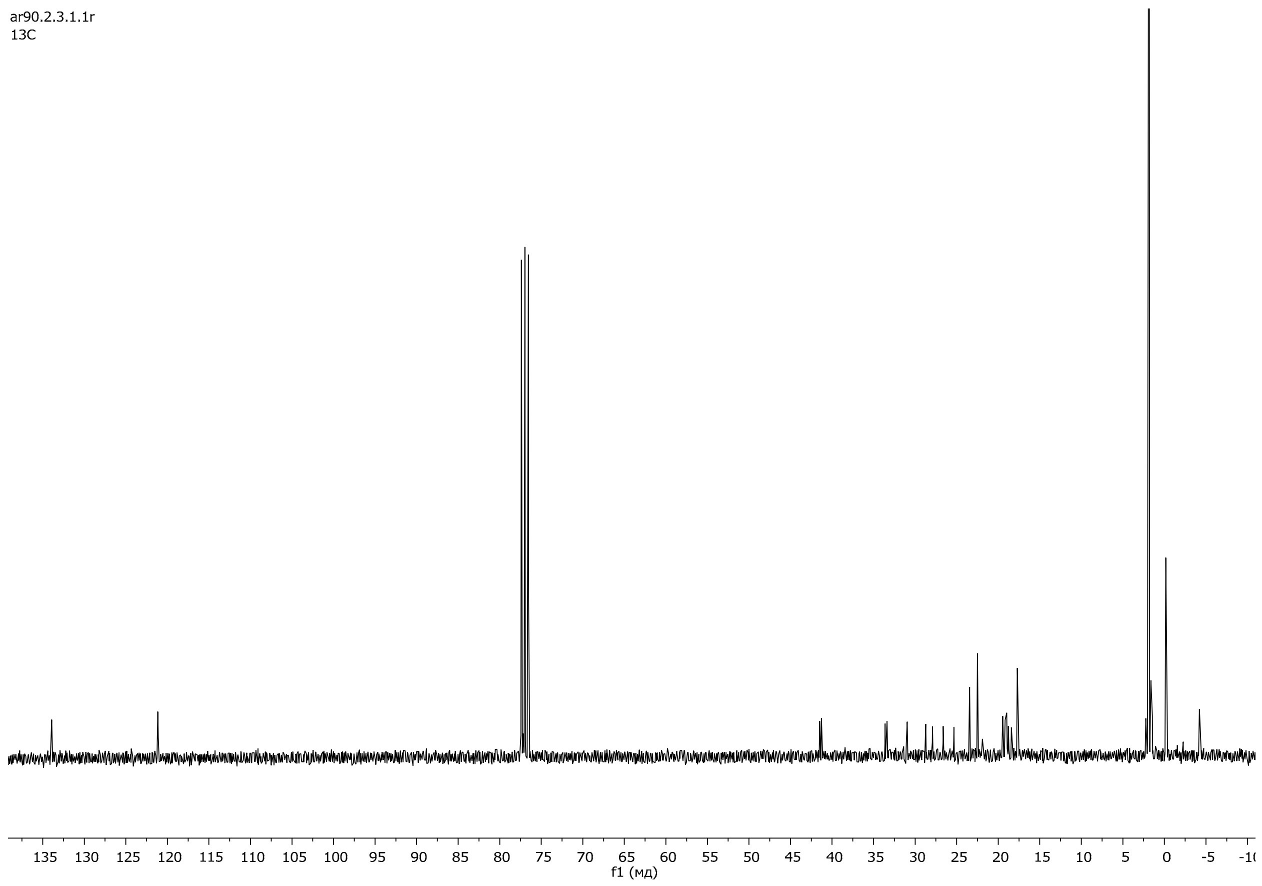

Figure S19. ^{13}C NMR spectrum of bis(heptamethylsilylpropyl)methylsilyllimonene (Lim-$G_{1,5}$TMS^{4}).

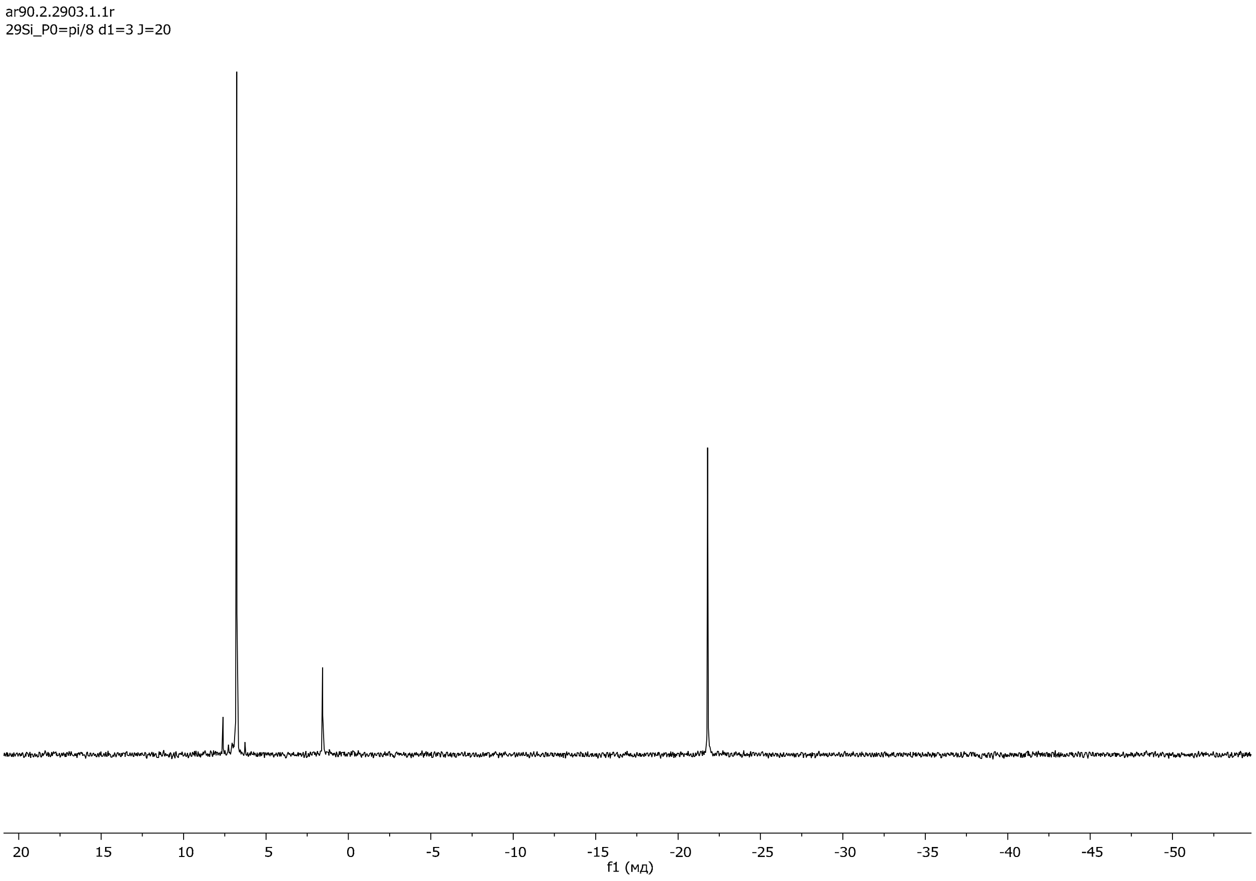

Figure S20. ^{29}Si NMR spectrum of bis(heptamethylsilylpropyl)methylsilyllimonene (Lim-$G_{1,5}TMS^4$).

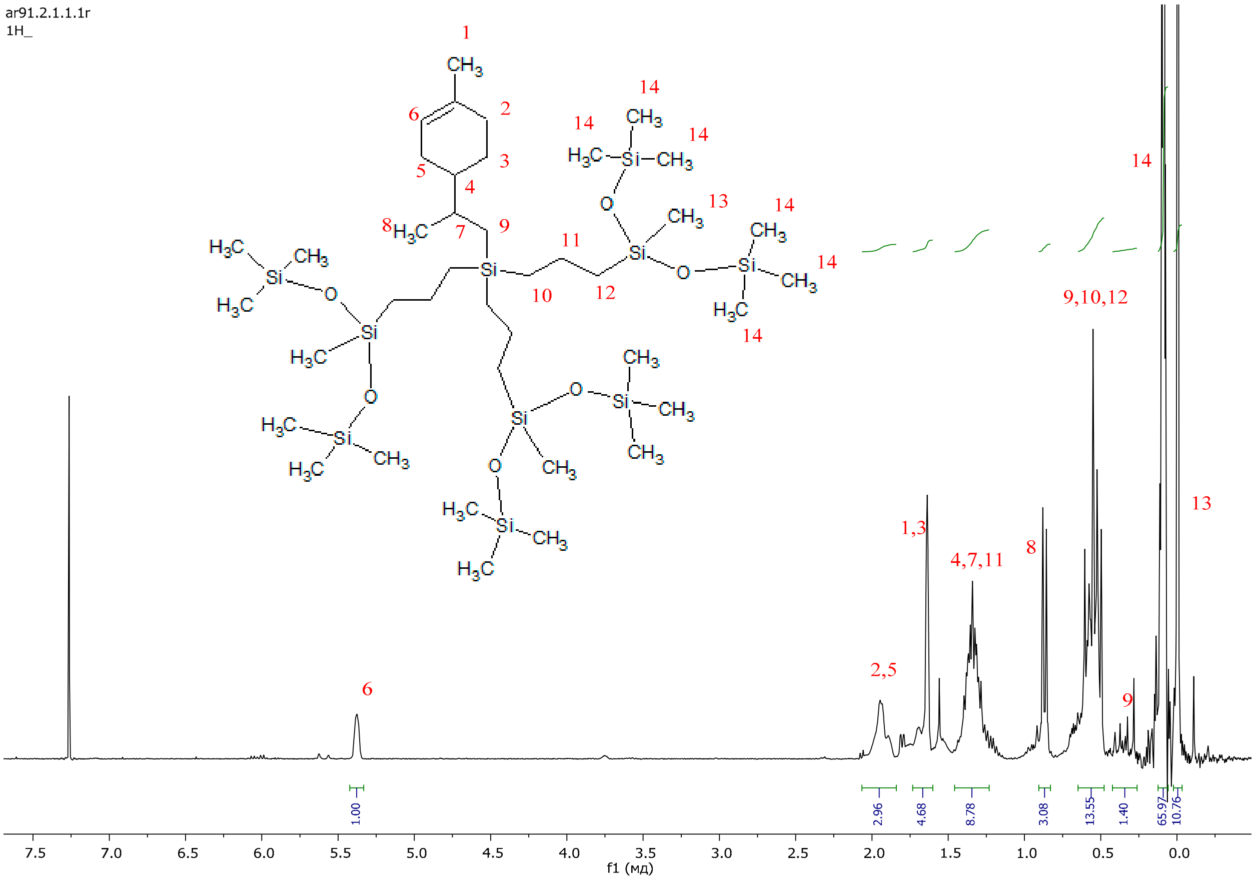

Figure S21. ^{1}H NMR spectrum of tris(heptamethylsilylpropyl)silyllimonene (Lim-G$_{1,5}$TMS^{6}).

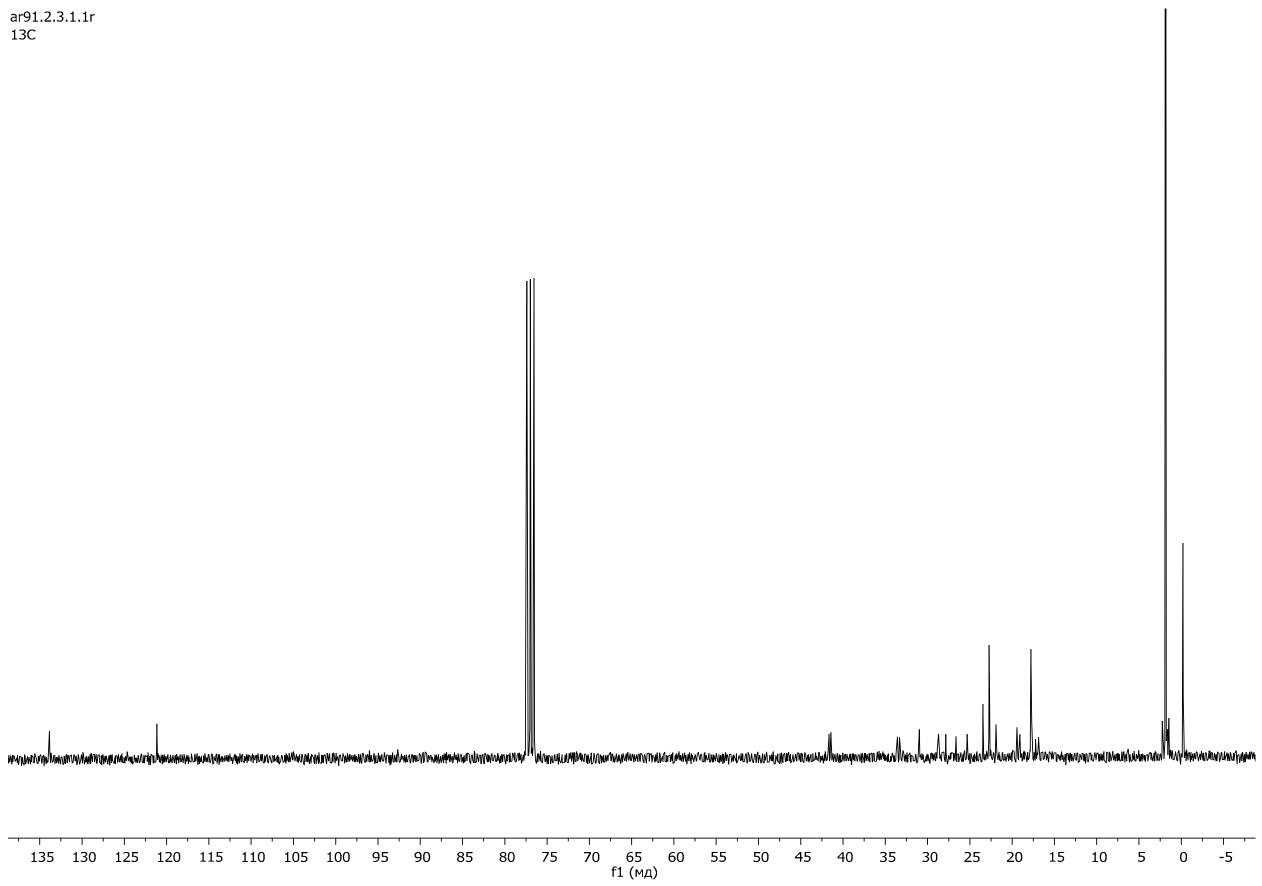

Figure S22. ^{13}C NMR spectrum of tris(heptamethylsilylpropyl)silyllimonene (Lim-$G_{1,5}$TMS^{6}).

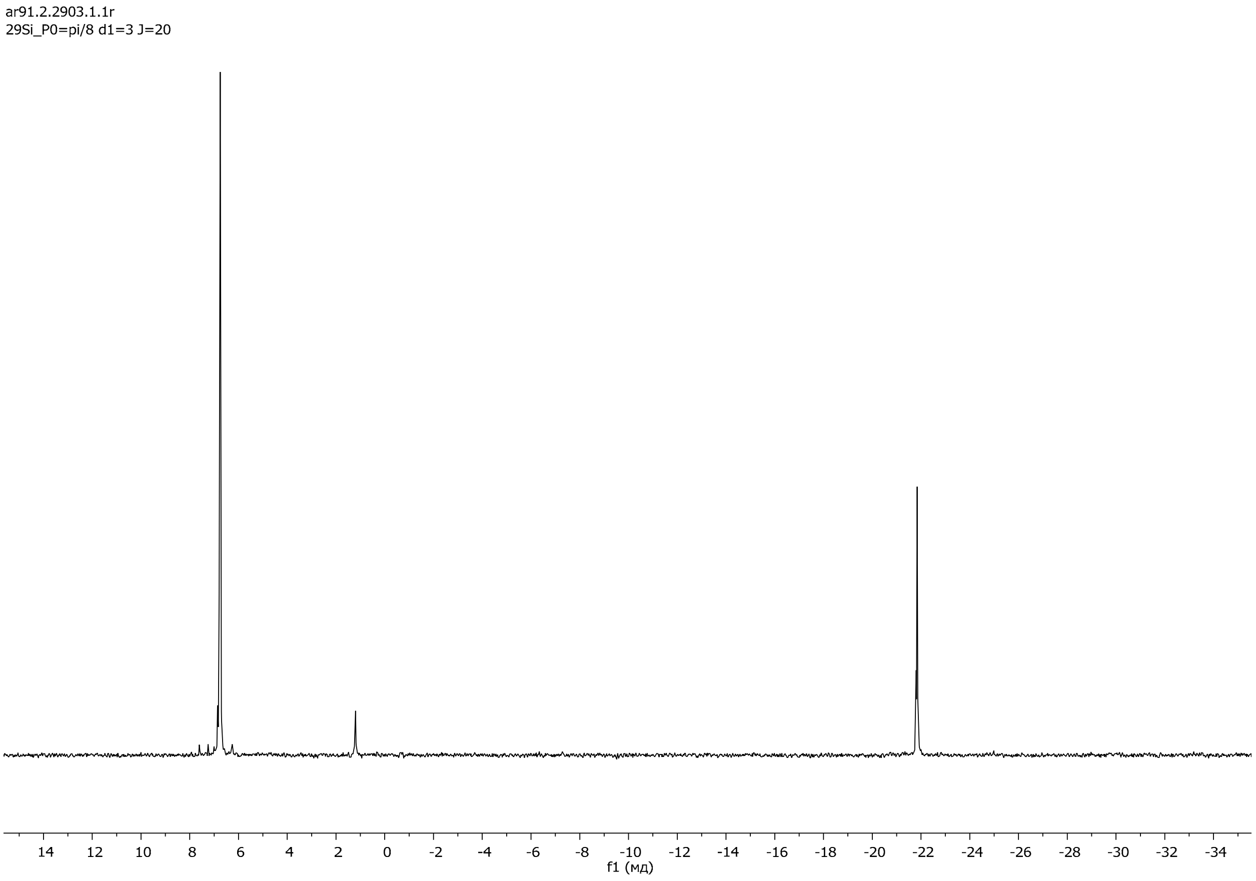

Figure S23. ^{29}Si NMR spectrum of tris(heptamethylsilylpropyl)silyllimonene (Lim-$G_{1,5}$TMS^{6}).

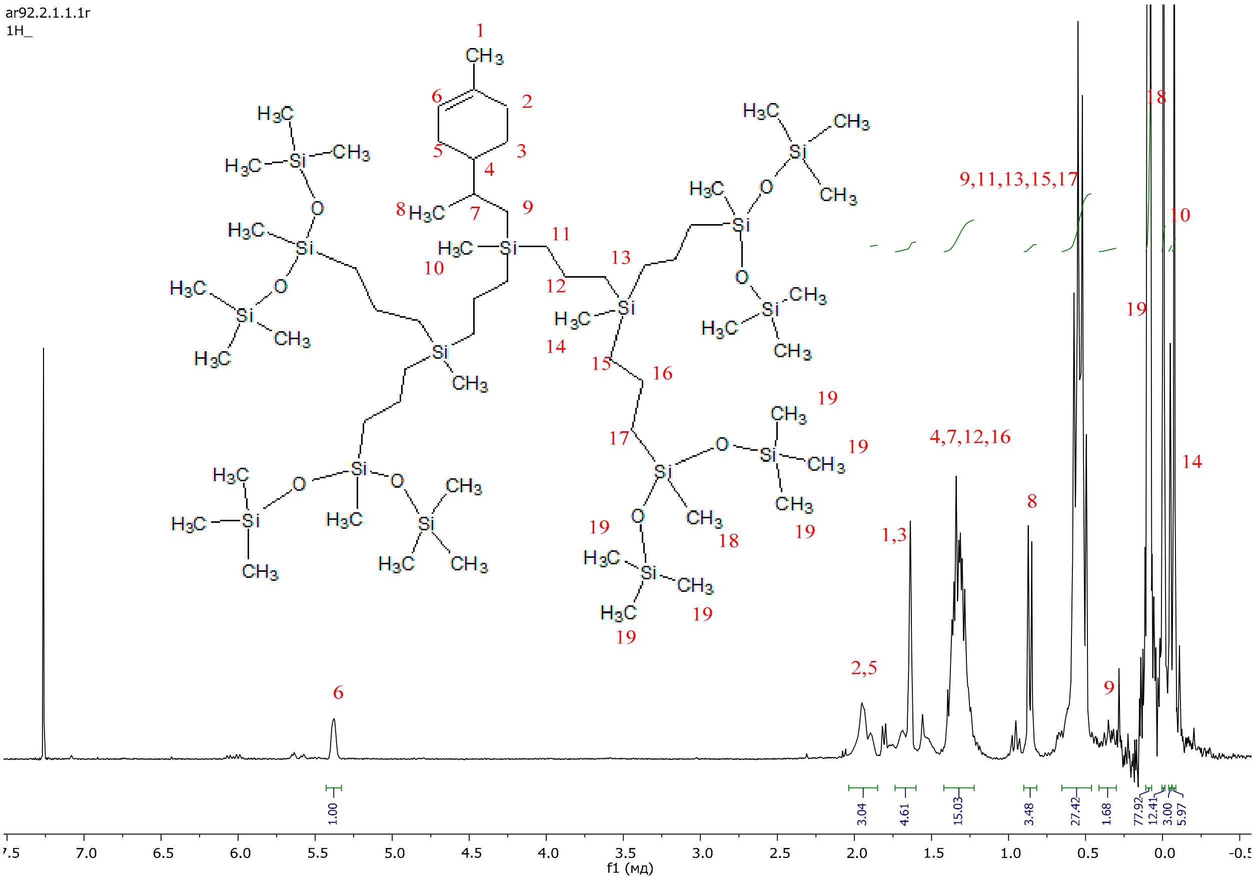

Figure S24. ^{1}H NMR spectrum of Lim-G$_{2,5}$TMS^{8}.

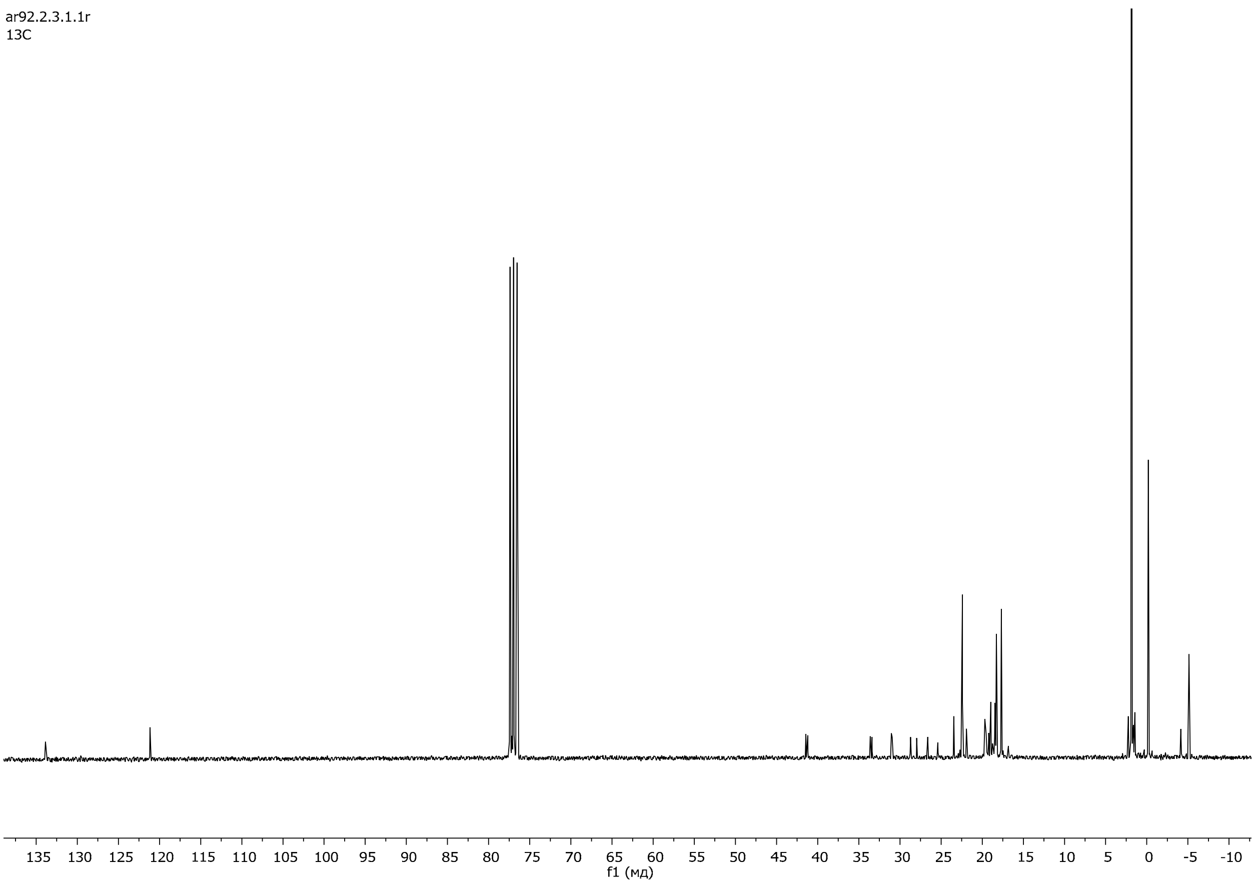

Figure S25. ^{13}C NMR spectrum of Lim-$G_{2,5}TMS^8$.

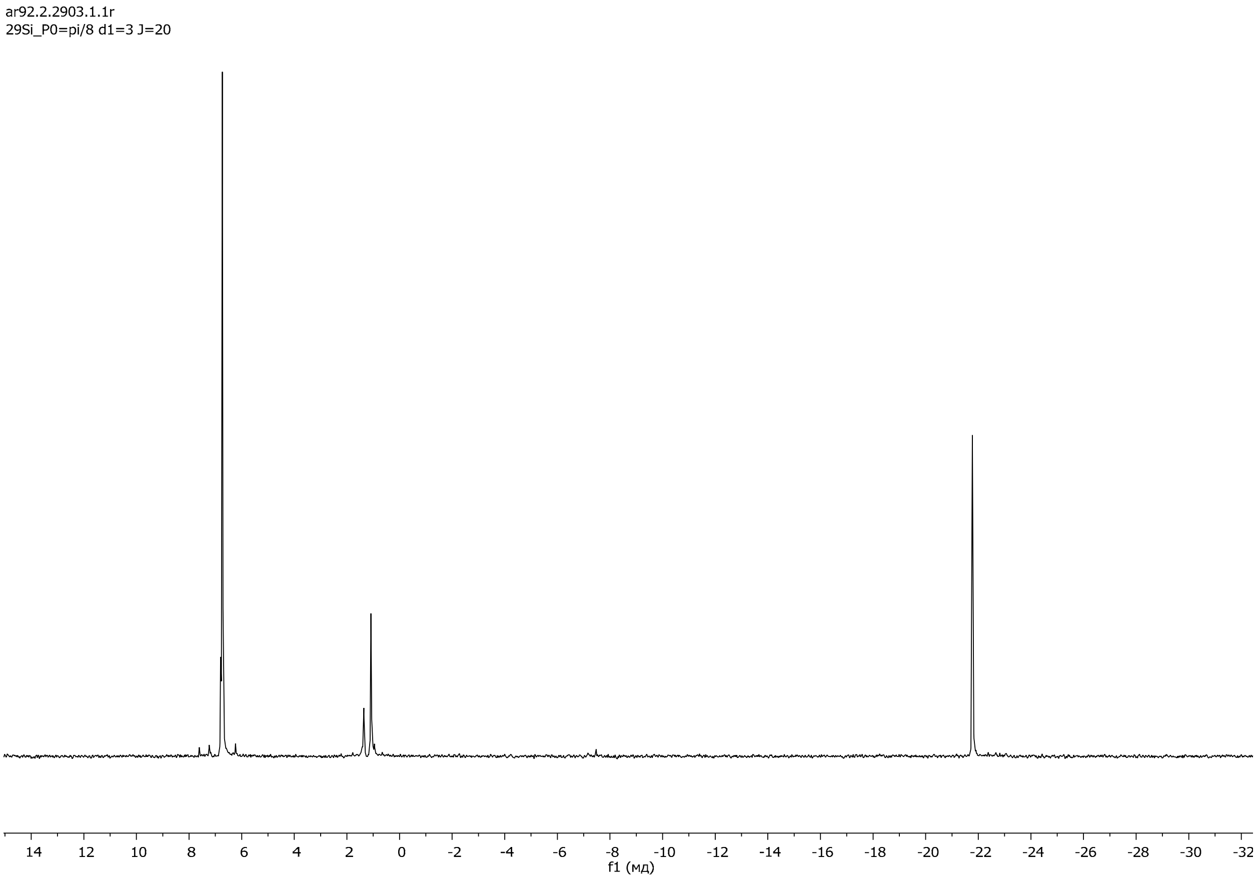

Figure S26. ^{29}Si NMR spectrum of Lim-$G_{2,5}$$TMS^8$.

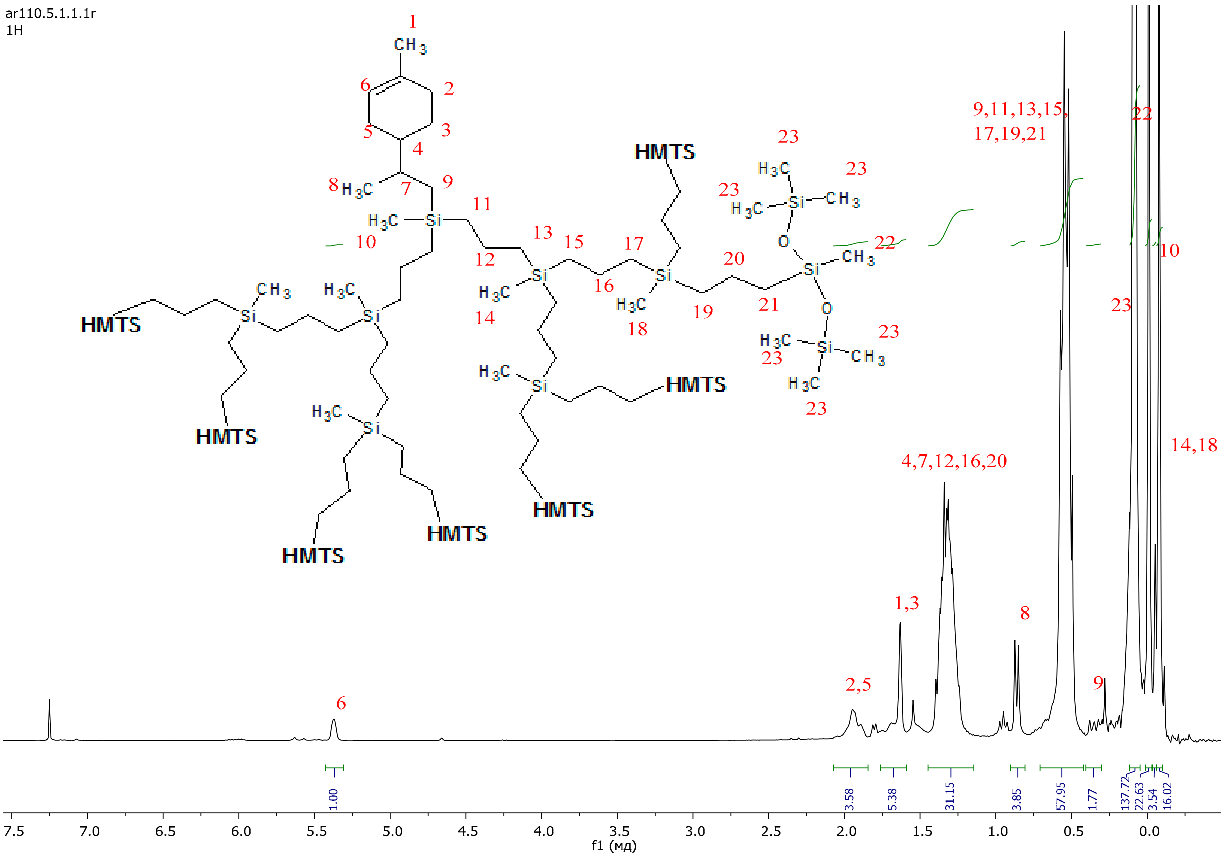

Figure S27. ^{1}H NMR spectrum of Lim-$G_{3,5}TMS^{18}$.

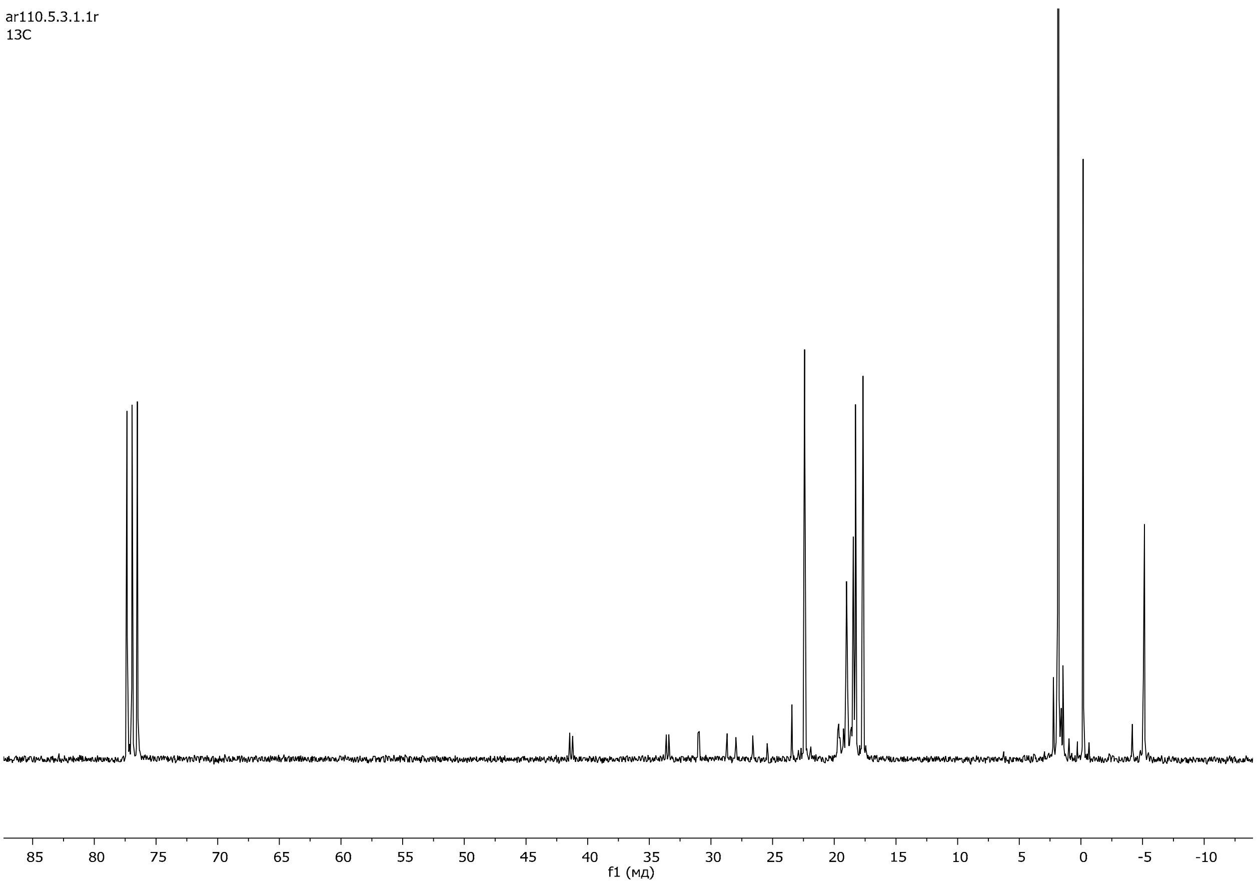

Figure S28. ^{13}C NMR spectrum of Lim-$G_{3,5}$TMS[18].

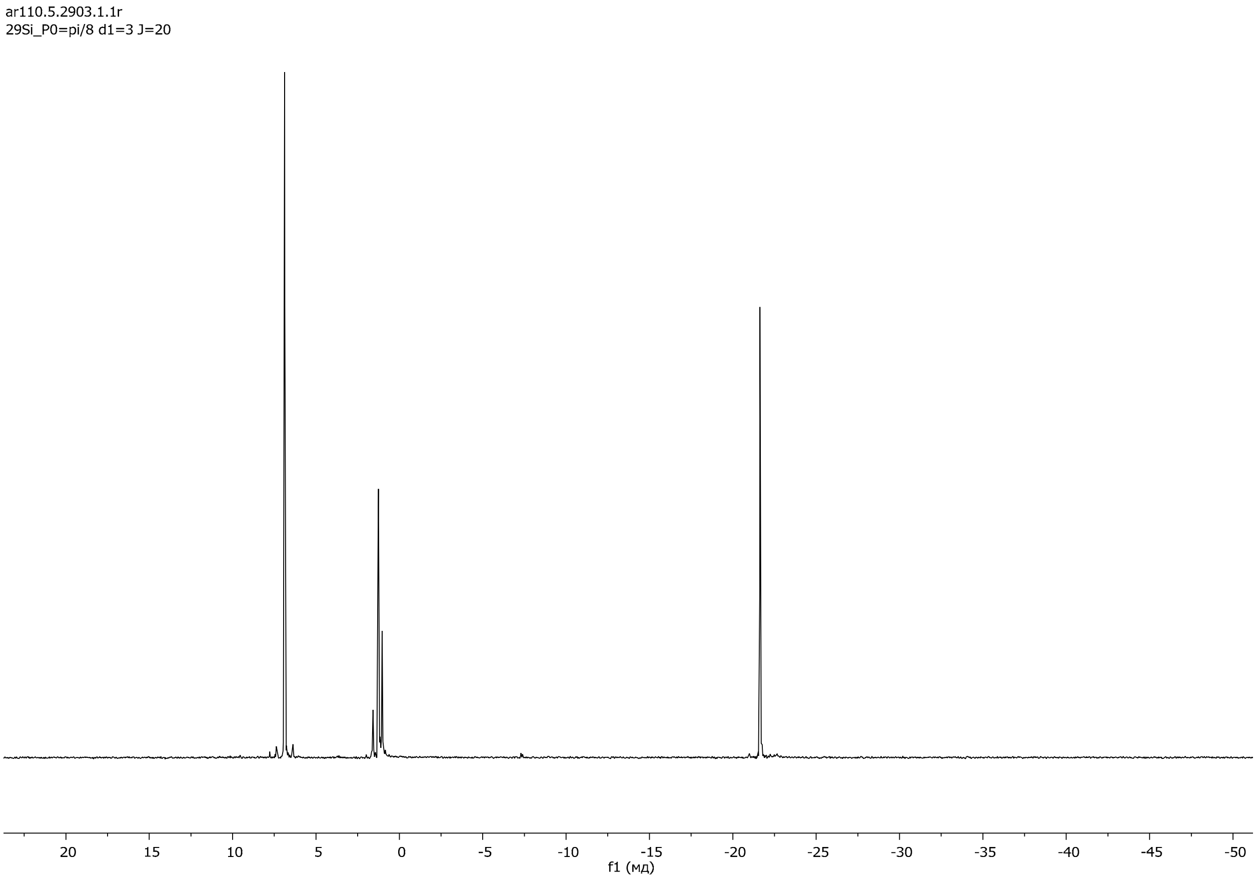

Figure S29. ^{29}Si NMR spectrum of Lim-$G_{3,5}$TMS[18].

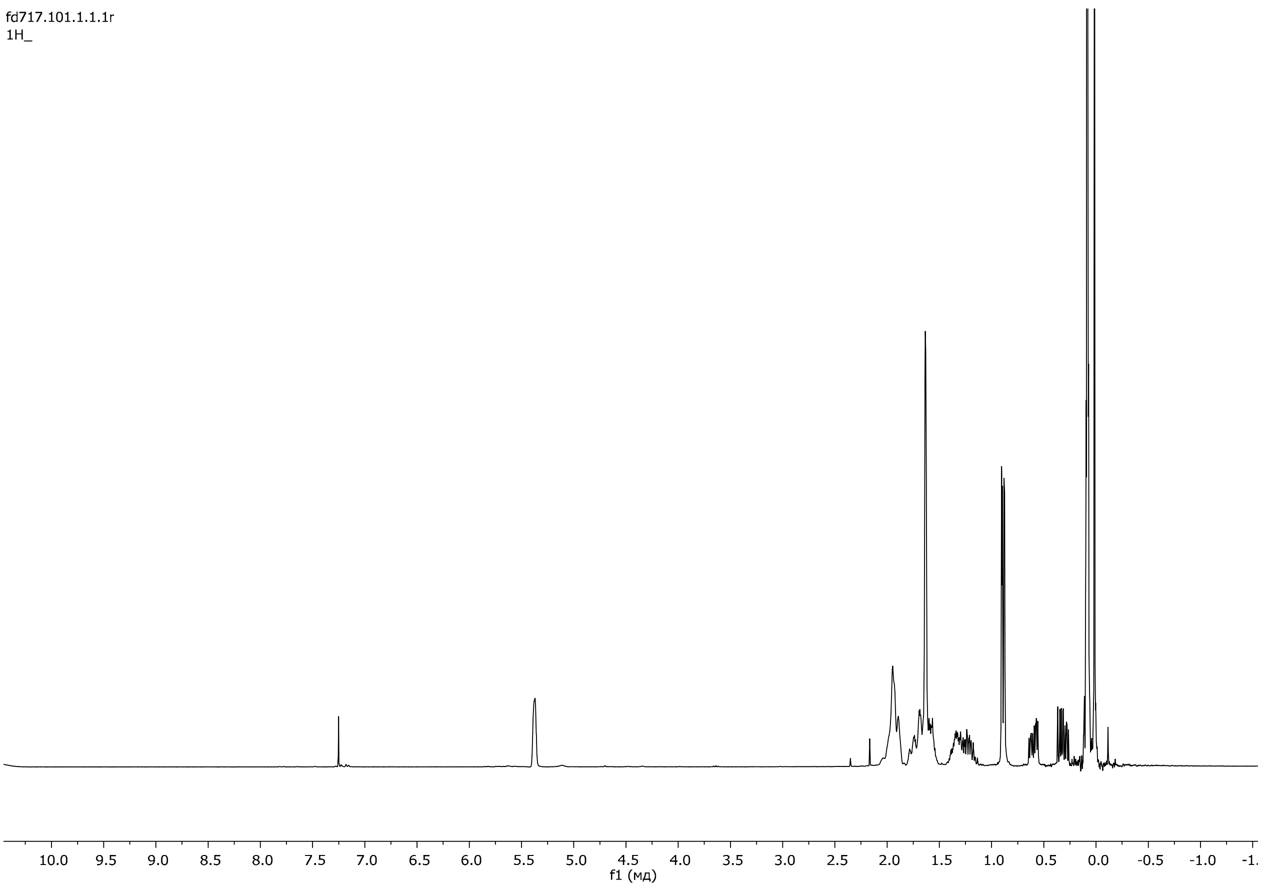

Figure S30. ^{1}H NMR spectrum of HMS-Lim.

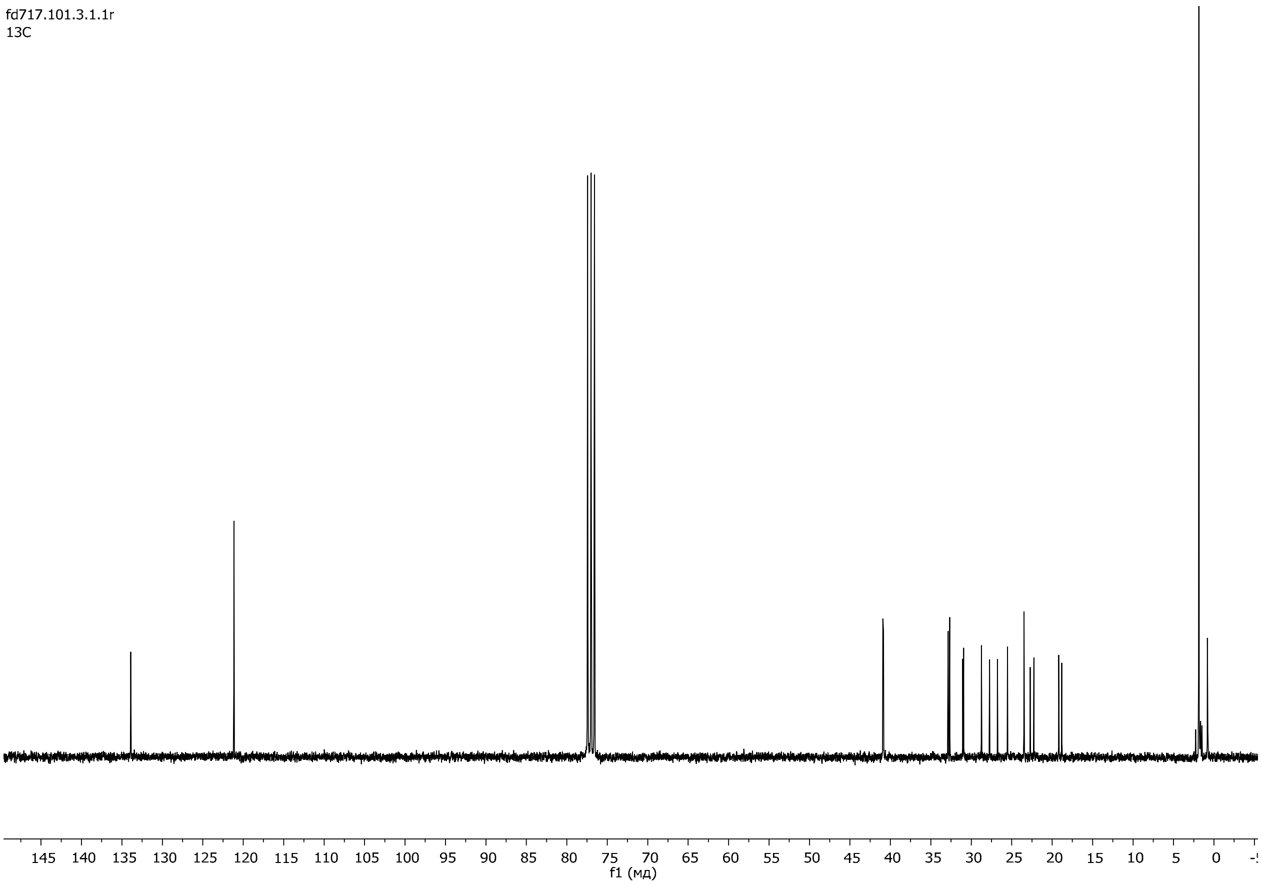

Figure S31. ^{13}C NMR spectrum of HMS-Lim.

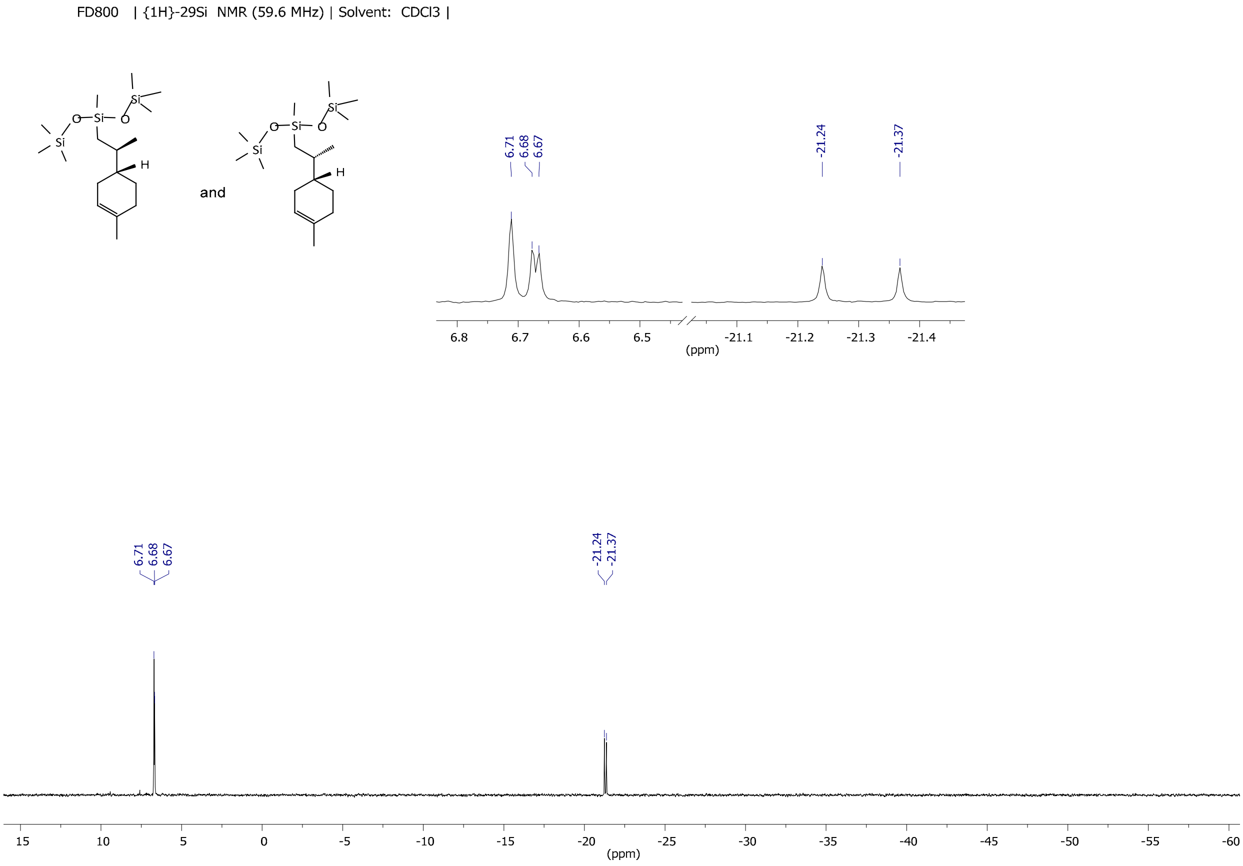

Figure S32. ^{29}Si NMR spectrum of HMS-Lim.

SI2. GPC curves

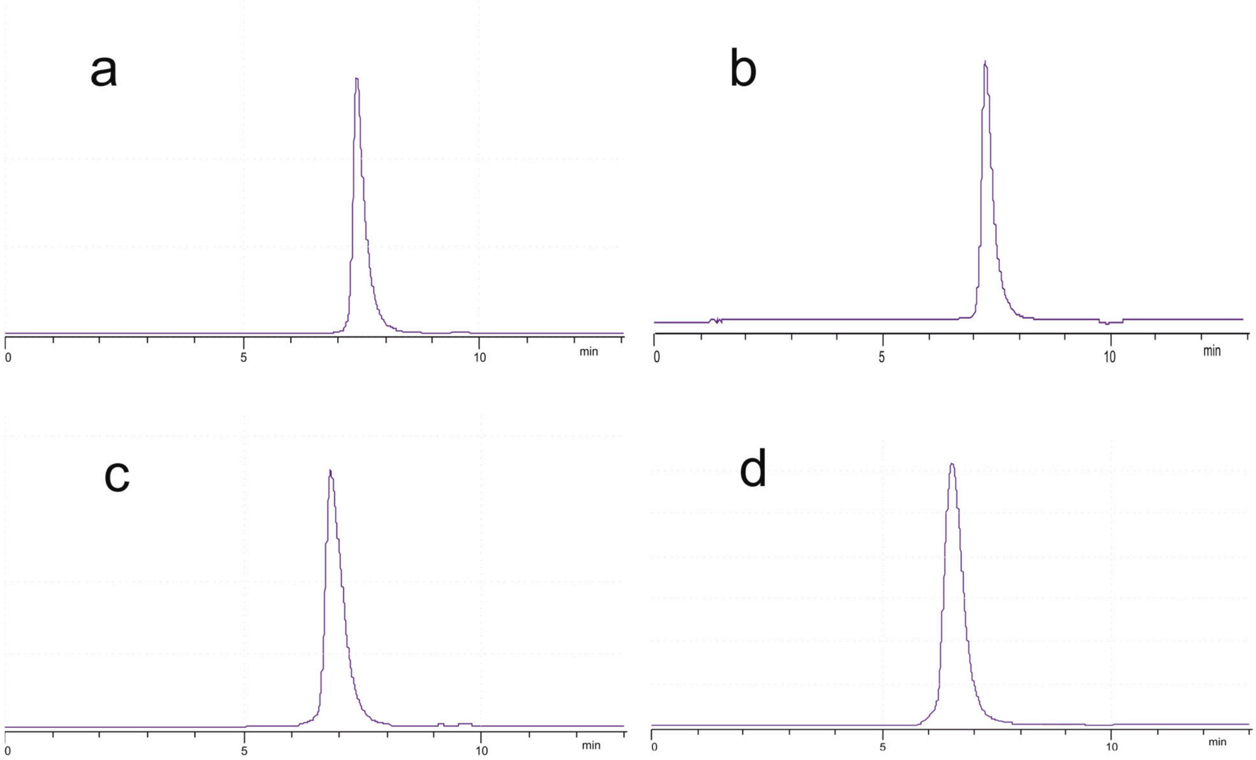

Figure S33. GPC chromatograms of dendrons based on limonene with allyl functionality on the periphery («a» - *Lim*-G_0All^2, «b» - *Lim*-G_0All^3, «c» - *Lim*-G_1All^4, «d» - *Lim*-G_2All^8).

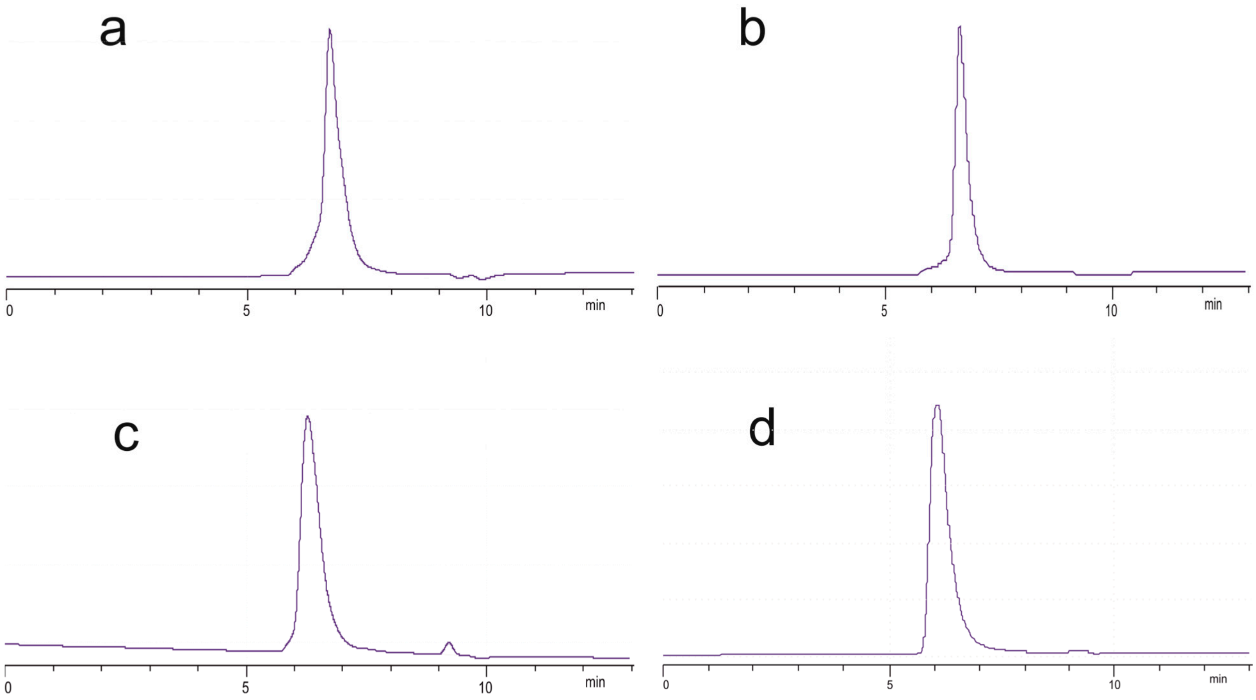

Figure S34. GPC chromatograms of dendrons based on limonene blocked with heptamethyltrisiloxane («a» - *Lim*-$G_{1,5}TMS^4$, «b» - *Lim*-$G_{1,5}TMS^6$, «c» - *Lim*-$G_{2,5}TMS^8$, «d» - *Lim*-$G_{3,5}TMS^{18}$).
